# Supplementary material for: An ancient pan-cnidarian microRNA regulates stinging capsule biogenesis in Nematostella vectensis
Source: Cell Rep. 2023 Sep 6;42(9):113072. doi: 10.1016/j.celrep.2023.113072 (PMC10548089; doi:10.1016/j.celrep.2023.113072)
Supplement: Document S1. Figures S1–S4 [file mmc1.pdf]

Cell Reports, Volume *42*

Supplemental information

An ancient pan-cnidarian microRNA regulates stinging capsule biogenesis in *Nematostella vectensis*

Arie Fridrich, Miguel Salinas-Saaverda, Itamar Kozlolvski, Joachim M. Surm, Eleni Chrysostomou, Abhinandan M. Tripathi, Uri Frank, and Yehu Moran

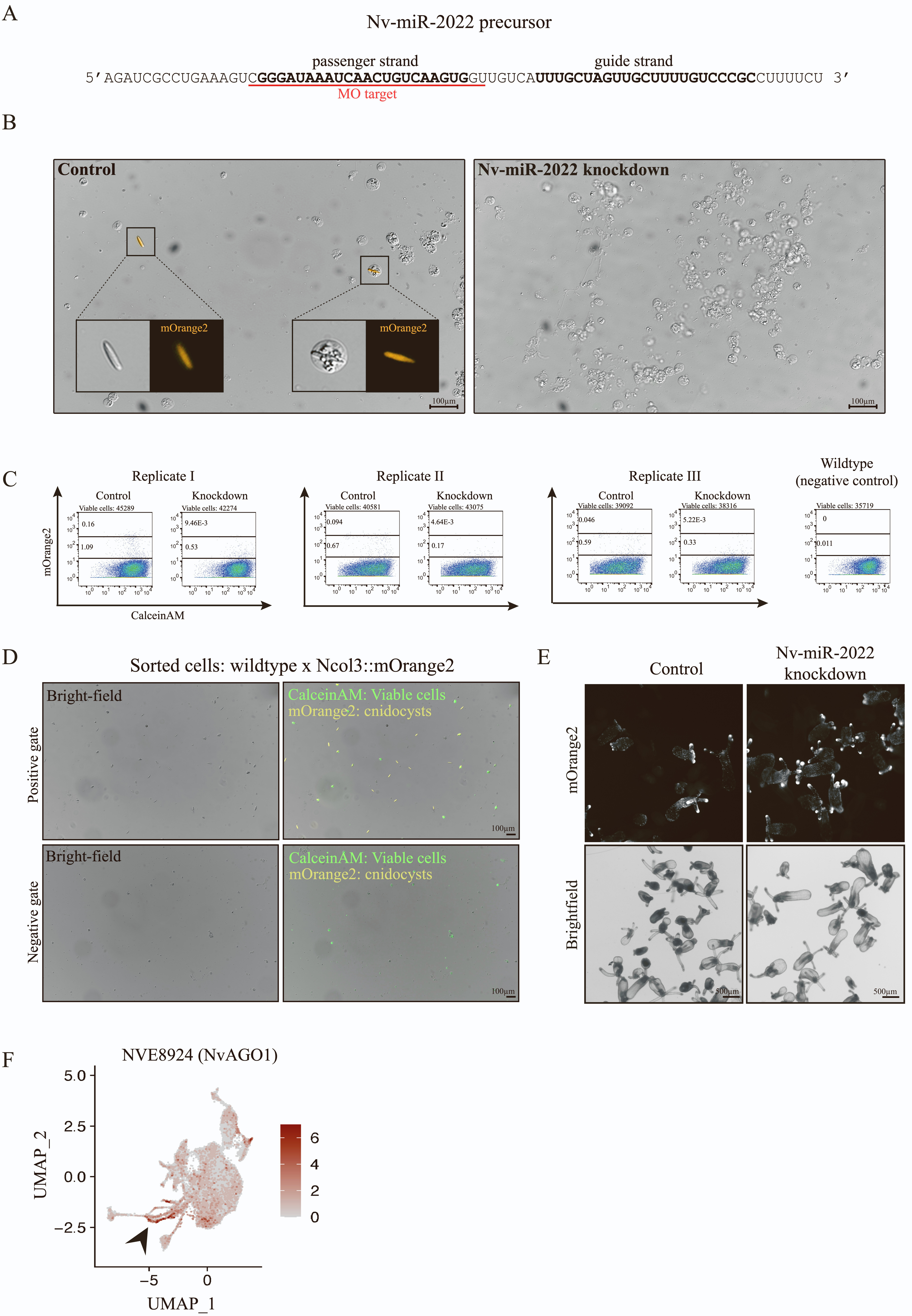
A
Nv-miR-2022 precursor
passenger strand
guide strand
5′AGAUCGCCUGAAAGUCGGGAUAAAUCAACUGUCAAGUGGUUGUCAUUUGCUAGUUGCUUUUGUCCCGCCUUUUCU 3′
MO target
B
Control
mOrange2
mOrange2
100μm
Nv-miR-2022 knockdown
100μm
C
Replicate I
Control
Knockdown
Viable cells: 45289
Viable cells: 42274
0.16
1.09
9.46E-3
0.53
Replicate II
Control
Knockdown
Viable cells: 40581
Viable cells: 43075
0.094
0.67
4.64E-3
0.17
Replicate III
Control
Knockdown
Viable cells: 39092
Viable cells: 38316
0.046
0.59
5.22E-3
0.33
Wildtype
(negative control)
Viable cells: 35719
0
0.011
mOrange2
CalceinAM
D
Sorted cells: wildtype x Ncol3::mOrange2
Positive gate
Negative gate
Bright-field
CalceinAM: Viable cells
mOrange2: cnidocysts
100μm
E
Control
Nv-miR-2022
knockdown
mOrange2
Brightfield
500μm
F
NVE8924 (NvAGO1)
UMAP_2
UMAP_1
5.0
2.5
0.0
−2.5
−5
0
6
4
2
0

Figure S1: The impact of miR-2022 knockdown on cnidogenesis and development of *Nematostella.* (**A**) Nv-miR-2022 precursor with highlighted region targeted by miR-2022 MO. (**B**) Dissociated cells of control and miR-2022 MO injected planulae 4 days post injection. Arrowheads mark cnidocytes. (**C**) Flow cytometry analysis of mOrange2::Ncol3 positive cells control and miR-2022 MO injected transgenes. Y axis represents the intensity of mOrange2 and cell viability is measured using CaleinAM on the X axis. Wildtype animals (right panel) were used set the threshold of the mOrange2 positive gate. Percentages of positive cells are indicates within the gates on the plot. (**D**) Cells from positive and negative gates were collected and visualized under a microscope to further confirm that glowing cells from the positive gates correspond to cnidocytes. (**E**) Intensity of mOrange2 and morphology of WT crossed with mOrange2::Ncol3 progeny 9 days post injecting the zygotes with control or miR-2022 MO. (**F**) Analysis of *Nematostella* single-cell RNA sequencing data generated by Steger, J. *et al.*[1] Arrowhead indicates cluster of cnidocytes and color intensity corresponds to expression intensities of NvAGO1.

A

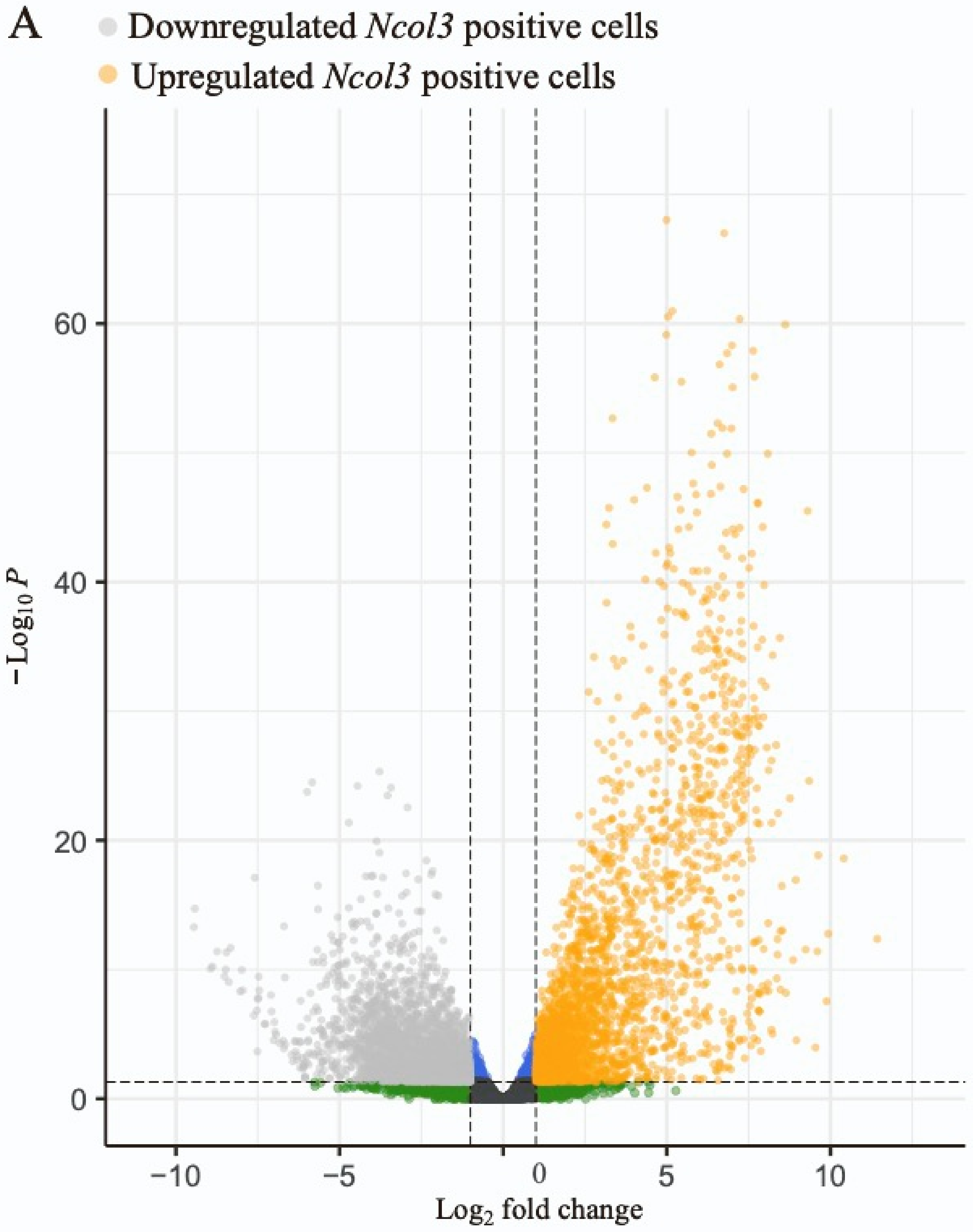
Downregulated *Ncol3* positive cells
Upregulated *Ncol3* positive cells
$-\mathrm{Log}_{10}\,P$
0
20
40
60
−10
−5
0
5
10
Log_2 fold change

Figure S2: differential expression analysis to Identify cnidocyte specific genes in *Nematostella* primary polyps of Ncol3::mOrange2 transgenic line (see methods for more details).

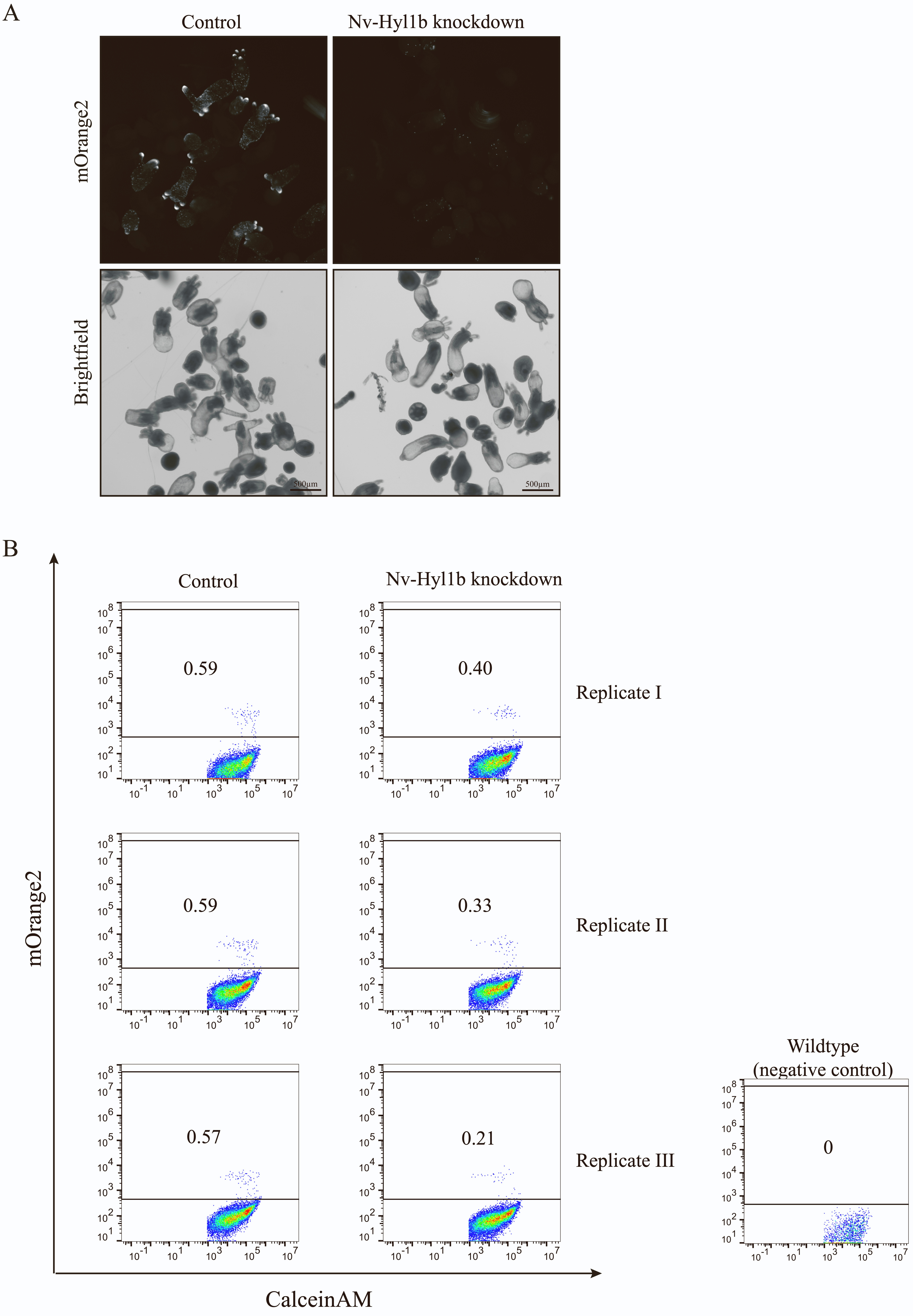

A
Control
Nv-Hyl1b knockdown
mOrange2
Brightfield
500μm
500μm
B
Control
Nv-Hyl1b knockdown
0.59
0.40
Replicate I
0.59
0.33
Replicate II
0.57
0.21
Replicate III
Wildtype
(negative control)
0
mOrange2
CalceinAM

Figure S3: The impact of Nv-Hyl1b knockdown on cnidogenesis and development of *Nematostella.* (**A**) Intensity of mOrange2 and morphology of WT crossed with mOrange2::Ncol3 progeny 9 days post injecting the zygotes with control or Nv-Hyl1b MO. (**B**) Flow cytometry analysis of mOrange2::Ncol3 positive cells control and Nv-Hyl1b MO injected transgenes. Y axis represents the intensity of mOrange2 and cell viability is measured using CaleinAM on the X axis. Wildtype animals (right panel) were used set the threshold of the memOrange2 positive gate. Percentages of positive cells are indicates within the gates on the plot.

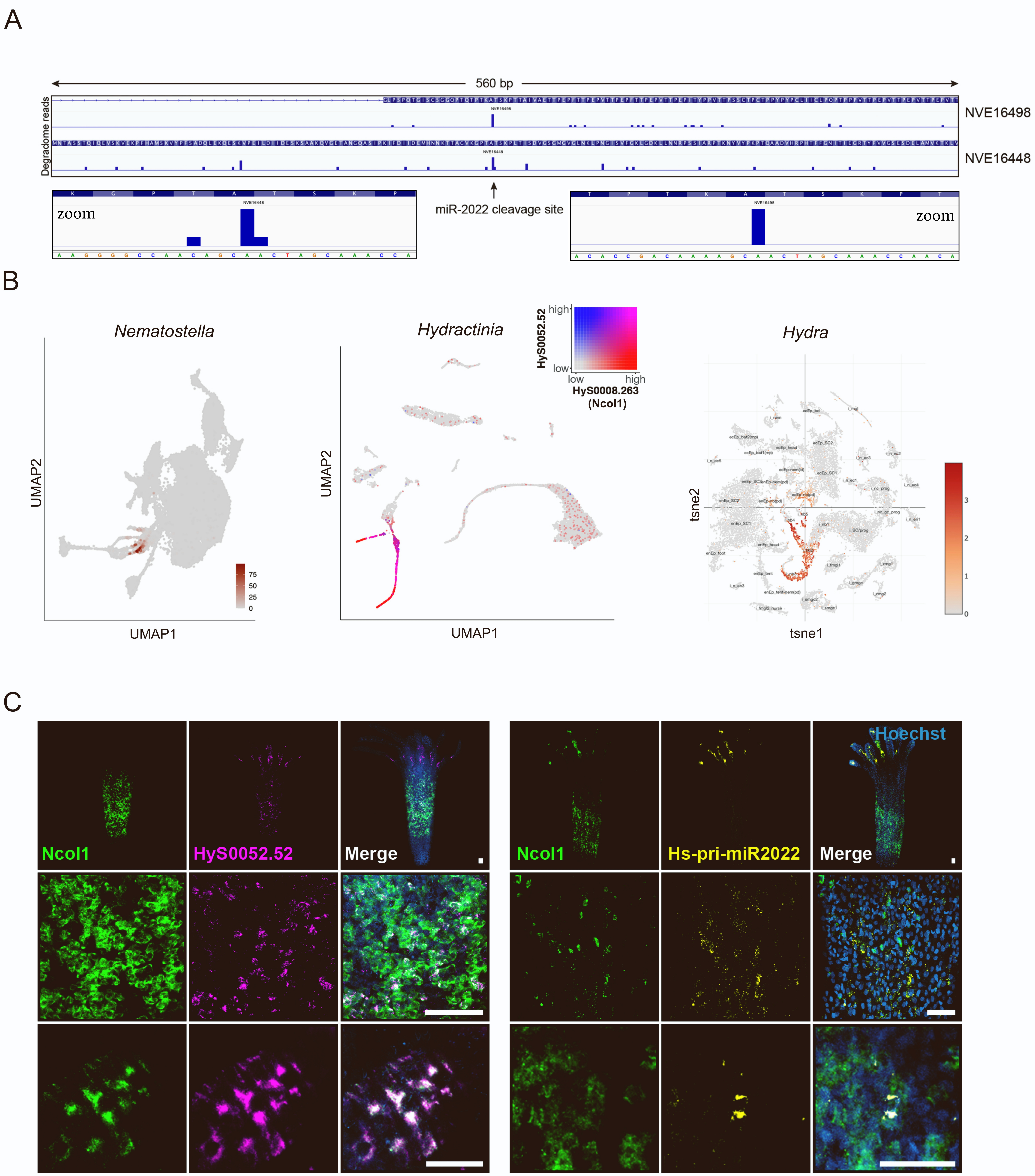
A
560 bp
Degradome reads
NVE16498
NVE16448
zoom
miR-2022 cleavage site
zoom
B
Nematostella
Hydractinia
Hydra
UMAP2
UMAP1
tsne2
tsne1
HyS0052.52
high
low
HyS0008.263
(Ncol1)
C
Ncol1
HyS0052.52
Merge
Ncol1
Hs-pri-miR2022
Merge
Hoechst

Figure S4: Expression of miR-2022 predicted targets in cnidocytes of *Hydra* and *Hydractinia*.(**A**) Degradome tracks spanning the experimentally validated Nv-miR-2022 targets NVE16498 and NVE16448 in *Nematostella*. (**B**) Single cells expressing NVE16448, Hys0052.52 and t19037aep in *Nematostella*, *Hydractinia* and *Hydra* (respectively). In *Hydractinia* expression intensities of Ncol1 and Hys0052.52 enable the visualization of the cnidocyte cluster of cells (bottom left) and the intensity of overlapping expression of Hys0052.52 within this cluster. In *Nematostella* the plot was generated as described in methods. In *Hydractinia* and *Hydra* plots were generated using the Single Cell Browser within the *Hydractinia* Genome Project Portal (https://research.nhgri.nih.gov/hydractinia) and using the *Hydra* single cell portal[2] (**C**) SABER FISH staining of Ncol1 (green) with Hys0052.52 (magenta) or with Hs-miR-2022 (yellow) in *Hydractinia* polyps.

1. Steger, J., Cole, A.G., Denner, A., Lebedeva, T., Genikhovich, G., Ries, A., Reischl, R., Taudes, E., Lassnig, M., and Technau, U. (2022). Single-cell transcriptomics identifies conserved regulators of neuroglandular lineages. Cell Rep *40*, 111370. 10.1016/j.celrep.2022.111370.
2. Siebert, S., Farrell, J.A., Cazet, J.F., Abeykoon, Y., Primack, A.S., Schnitzler, C.E., and Juliano, C.E. (2019). Stem cell differentiation trajectories in Hydra resolved at single-cell resolution. Science *365*. 10.1126/science.aav9314.
