# Supplementary material for: An ancient pan-cnidarian microRNA regulates stinging capsule biogenesis in Nematostella vectensis
Source: Cell Rep. 2023 Sep 6;42(9):113072. doi: 10.1016/j.celrep.2023.113072 (PMC10548089; doi:10.1016/j.celrep.2023.113072)
Supplement: Document S2. Article plus supplemental information [file mmc6.pdf]

Cell Reports

Report

An ancient pan-cnidarian microRNA regulates stinging capsule biogenesis in *Nematostella vectensis*

Graphical abstract

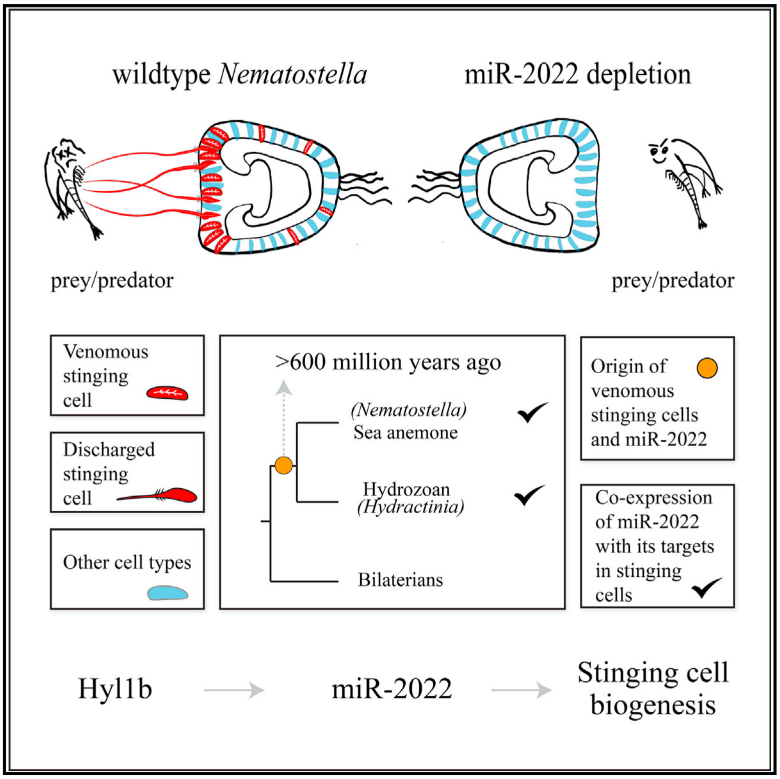

Authors

Arie Fridrich, Miguel Salinas-Saaverda, Itamar Kozlolvski, ..., Abhinandan M. Tripathi, Uri Frank, Yehu Moran

Correspondence

arie.fridrich@gmi.oeaw.ac.at (A.F.), yehu.moran@mail.huji.ac.il (Y.M.)

In brief

Fridrich et al. unravel a role for miR-2022 in the formation of cnidocytes, ancient cnidarian-specific cells. By manipulating miR-2022 levels in *Nematostella*, they demonstrate it is essential for cnidocyte biogenesis and prey paralysis. The conservation of miR-2022 across all major cnidarian classes suggests its contribution to this phylum's evolutionary success.

Highlights

- miR-2022 is crucial for formation of the cnidocyte, an ancient complex cell type in Cnidaria
- Manipulating miR-2022 affects cnidocyte biogenesis and prey/predator paralysis
- Conservation of miR-2022 suggests its functional significance across diverse cnidarian species

Fridrich et al., 2023, Cell Reports *42*, 113072
September 26, 2023 © 2023 The Authors.
https://doi.org/10.1016/j.celrep.2023.113072

CellPress

Cell Reports

CellPress
OPEN ACCESS

Report

An ancient pan-cnidarian microRNA regulates stinging capsule biogenesis in *Nematostella vectensis*

Arie Fridrich,[1,3,*] Miguel Salinas-Saaverda,[2] Itamar Kozlolvski,[1] Joachim M. Surm,[1] Eleni Chrysostomou,[2] Abhinandan M. Tripathi,[1] Uri Frank,[2] and Yehu Moran[1,4,*]
[1]Department of Ecology Evolution and Behavior, Faculty of Science, Alexander Silberman Institute of Life Sciences, The Hebrew University of Jerusalem, Jerusalem, Israel
[2]Centre for Chromosome Biology, University of Galway, Galway, Ireland
[3]Gregor Mendel Institute of Molecular Plant Biology, Austrian Academy of Sciences, Vienna, Austria
[4]Lead contact
*Correspondence: arie.fridrich@gmi.oeaw.ac.at (A.F.), yehu.moran@mail.huji.ac.il (Y.M.)
https://doi.org/10.1016/j.celrep.2023.113072

SUMMARY

An ancient evolutionary innovation of a novel cell type, the stinging cell (cnidocyte), appeared >600 million years ago in the phylum Cnidaria (sea anemones, corals, hydroids, and jellyfish). A complex bursting nano-injector of venom, the cnidocyst, is embedded in cnidocytes and enables cnidarians to paralyze their prey and predators, contributing to this phylum's evolutionary success. In this work, we show that post-transcriptional regulation by a pan-cnidarian microRNA, miR-2022, is essential for biogenesis of these cells in the sea anemone *Nematostella vectensis*. By manipulation of miR-2022 levels in a transgenic reporter line of cnidocytes, followed by transcriptomics, single-cell data analysis, prey paralysis assays, and cell sorting of transgenic cnidocytes, we reveal that miR-2022 enables cnidocyte biogenesis in *Nematostella*, while exhibiting a conserved expression domain with its targets in cnidocytes of other cnidarian species. Thus, here we revealed a functional basis to the conservation of one of nature's most ancient microRNAs.

INTRODUCTION

MicroRNAs (miRNAs) are small non-coding RNAs of 20–24 nucleotides that regulate messenger RNA (mRNA) levels in plants and animals.[1–3] In bilaterians (animals with bilateral symmetry such as arthropods, nematodes, and vertebrates) these molecules are loaded into Argonaute (AGO) proteins and bind mRNAs through a partial base pair complementarity[4] (~7 nucleotides at positions 2–8 of the miRNA, known as "seed"). Consequently, the RNA-induced silencing complex (RISC) and the AGOs at their core are guided by the miRNA to reduce target mRNA levels as well as promoting translational inhibition.[2] This enables the regulation of cell differentiation and developmental processes. Among animals, the importance of individual miRNAs has been demonstrated in common lab model organisms belonging to the Bilateria group.[5–8]

Cnidarias (sea anemones, corals, hydroids, and jellyfish) comprise a sister group that evolved in parallel to bilaterians for over 600 million years with a different set of their own microRNAs. Cnidarian miRNAs exhibit an ancestral mode of action as they bind their mRNA target with high complementarity (similarly to plants) to mediate target cleavage by the catalytic domain of AGOs.[9] In addition, homologs of an ancestral miRNA biogenesis factor, which was considered plants specific (hyponastic leaves 1 [Hyl1]), were found in cnidarians but not in bilaterians.[10,11] miRNA turnover is higher in cnidarians relatively to bilaterians.[9,12] Yet, miR-2022 is one of the most conserved metazoan miRNAs and is found in all genomes of cnidarians sequenced to date.[9,12–16] Thus, it is one of only two pan-cnidarian miRNAs. However, miR-2022 function has remained unknown since its discovery in 2008.[12] In the sea anemone *Nematostella vectensis*, miR-2022 is expressed in cnidocytes.[9]

Cnidocytes are a cnidarian defensive/offensive cell-type innovation that originated from the neurosecretory lineage[17–19] >600 million years ago after the divergence from Bilateria. This cell lineage is essential for members of this ancient phylum to capture prey and repel predators through biogenesis of the cnidocyst: a stinging organelle that explosively discharges and either entangles or injects the prey or predator with venom.[20,21] The cnidocyst is one of the most complex biological structures in nature and its discharge is one of the fastest known biomechanical processes.[22–24]

While extensive efforts were devoted to identification of structural and venomous components of these organelles,[20,25–32] little is known about the regulatory mechanisms that enable their biogenesis beyond transcription factors. Nematogalectin-related 2 (NR2) is a structural component of the nematocyst tubule that is targeted by and co-expressed with miR-2022 in *Nematostella*.[9]

This is an open access article under the CC BY license (http://creativecommons.org/licenses/by/4.0/).

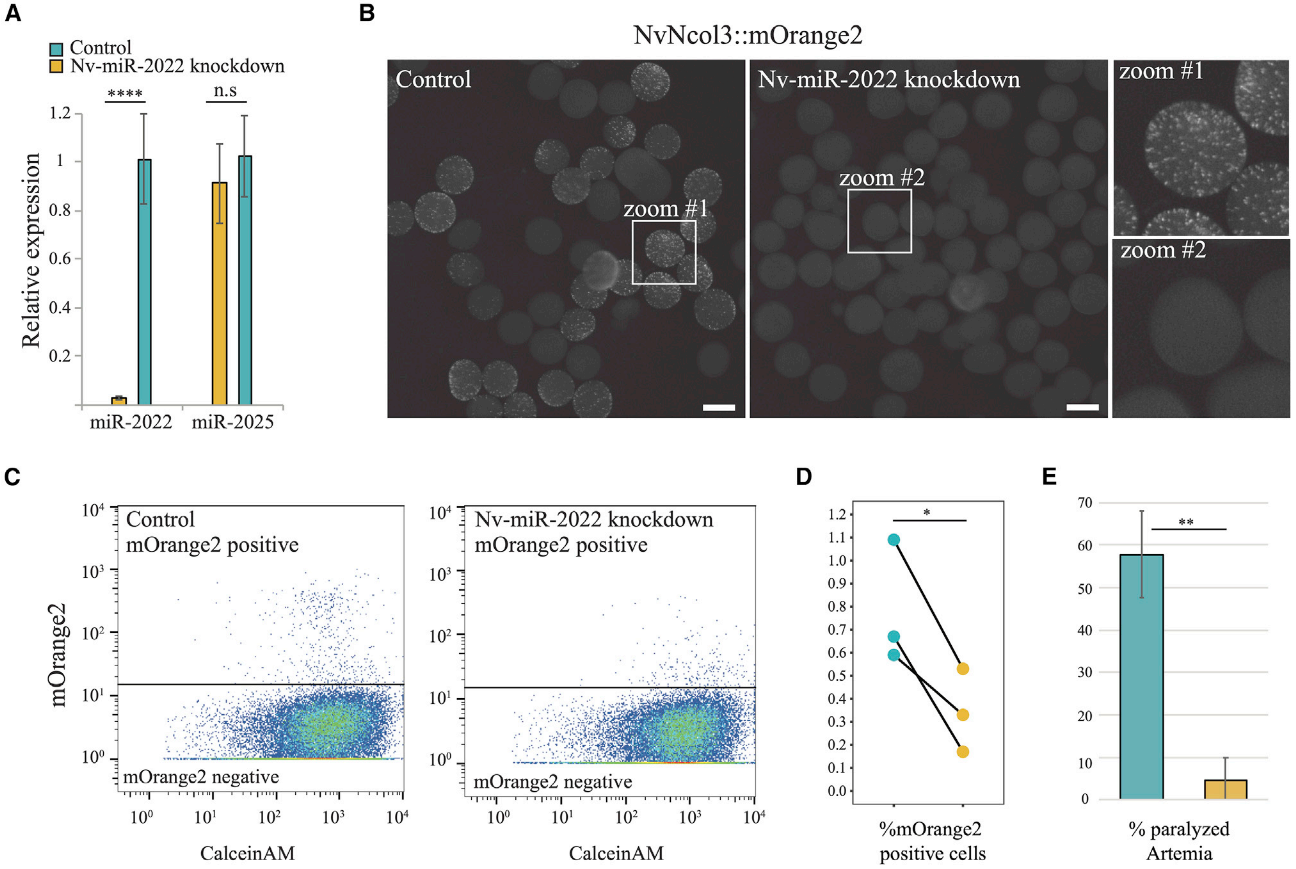

Figure 1. The impact of miR-2022 knockdown on cnidogenesis and prey paralysis potency in *Nematostella*

(A) Stem-loop qPCR validation of Nv-miR-2022 levels in *Nematostella* planulae 4 days post injection of control or miR-2022 MO into zygotes. Three independent biological replicates. Student's t test. p = 1.87E−11. Error bars represent standard deviation.

(B–D) (B) Intensity of mOrange2 in progeny of wild type (WT) crossed with mOrange2::Ncol3 transgenes 4 days post injecting the zygotes with control or miR-2022 MO. Scale bars, 200 μm. Three independent biological replicates were subjected to flow cytometry analysis and the percentage of mOrange2-positive cells was quantified (C and D).

(E) Percentage of paralyzed *Artemia* 5.5 h post incubation with control or miR-2022 MO 4-day-old *Nematostella* planulae (three independent biological replicates). Student's t test. p = 0.009489. Error bars represent standard deviation.

Notably, the functional role of individual miRNAs in animals was never explored in non-bilaterians. Thus, in this work, we tested the role of miR-2022 and the functional basis for its conservation across 600 million years of cnidarian evolution.

RESULTS AND DISCUSSION

miR-2022 contributes to biogenesis of cnidocytes and fitness of *Nematostella*

miRNA precursors are stem-loop structures that are recognized by miRNA biogenesis factors such as Dicer and Drosha.[33] To test if Nv-miR-2022 takes part in the biogenesis of cnidocytes we designed a morpholino antisense oligo (MO) to interfere with Nv-miR-2022 biogenesis by binding the pri-miR and hence masking Dicer and Drosha processing sites[34] (Figure S1A). Knockdown specificity was validated using stem-loop real-time quantitative PCR (qPCR)[35] four days post injection of the MO to *Nematostella* zygotes (Figure 1A). Wild-type (WT) females were crossed with a transgenic male that expressed the fluorescent protein memOrange2 under the regulation of the promoter of the cnidocyte-specific gene *NvNcol-3* (Ncol3::memOrange2[28]). As a result, ∼50% of the progeny exhibited fluorescently labeled cnidocytes (Figure 1B, left panel). After injecting the zygotes of WT/Ncol3::memOrange2 cross with Nv-miR-2022-MO, we visually detected an overall reduction of fluorescent cnidocytes (Figure 1B). While the overall percentage of cnidocytes in *Nematostella* is low during the first few days of development (∼2% of all cells[36]), we could easily detect cnidocyst capsules among dissociated cells of control injected animals in contrast to animals injected with miR-2022-MO (Figure S1B). Next, dissociated cells of miR-2022 morphants were subjected to flow cytometry analysis, which enabled us to quantify a significant reduction in the number of fluorescently labeled cnidocytes (Figures 1C, 1D, S1C, and S1D). Notably, the fraction of highly fluorescent cnidocytes was more reduced than the fraction composed of weaker expression (Figure S1C).

We have previously shown that global inhibition of the miRNA pathway by knocking down key biogenesis factors in *Nematostella* results in severe developmental defects, which prevents the metamorphosis from the swimming planulae stage to sessile polyps.[11,37,38] However, the knockdown of miR-2022 did not impact this developmental transition and 9 days post injection we observed metamorphosed polyps (Figure S1E). Thus, we conclude that miR-2022 plays a vital and specific role in the regulation of cnidocyte biogenesis. *Nematostella* encodes two paralogs of AGO proteins: NvAGO1 and NvAGO2. Interestingly, while NvAGO1 can be found in diverse cell types, it exhibits a strong enrichment in the cluster that corresponds to cnidocytes in *Nematostella* single-cell RNA sequencing data[19] (Figure S1F). Using NveAGO1 and NveAGO2 immunoprecipitations, we previously showed that miR-2022 exhibits the highest preference to load into NvAGO1,[37] demonstrating the overall regulatory importance of the miRNA pathway in cnidocytes.

Next, we hypothesized that the defensive capabilities of morphants of Nv-miR-2022 would be reduced due to the reduction in mature cnidocytes. Previously we showed the defensive capability of young *Nematostella* larvae through stinging potential predators.[27] We utilized a similar assay and incubated 4-day-old *Nematostella* planulae, which were injected with either control or Nv-miR-2022 MO, with nauplii of the brine shrimp *Artemia salina*. At this developmental stage, prior to metamorphosis, *Nematostella* is not capable of feeding on *Artemia*. Therefore, successful paralysis represents defensive capabilities. Five and a half hours post-incubation, *Artemia* incubated with control injected *Nematostella* showed a significantly higher number of paralyzed/dead individuals compared with *Artemia* incubated with Nv-miR-2022 MO-injected *Nematostella* (t test, $p = 0.009489$, Figure 1E; Video S1).

The loss of cnidocytes in *Nematostella* miR-2022 morphants as well as their reduced potency in paralyzing *Artemia*, yet without impacting the developmental metamorphosis of the animal, suggest that miR-2022 carries a specific role to regulate a restricted cell-type population that is vital for the survival of cnidarians.

To further explore the specificity of miR-2022 function in cnidocytes we measured gene expression levels in *Nematostella* miR-2022 morphants.

Transcriptome of *Nematostella* miR-2022 morphants is enriched with mis-regulated cnidocyte-specific genes

To assay the effect of Nv-miR-2022 knockdown on gene expression we extracted RNA from 4-day-old Nv-miR-2022 morphants and animals injected with control MO and employed high-throughput RNA sequencing. We carried out differential expression analysis using DESeq2,[39] which enabled us to identify 944 differentially expressed genes (DEGs). The majority of DEGs in miR-2022 morphants appeared to be downregulated (805 of 944) (Figure 2A). Recently, our NvNcol3::memOrange2 transgenic line was utilized to isolate the cnidocytes of *Nematostella* primary polyps and profile their transcriptome.[40] We re-analyzed these transcriptomes and generated a list of 2,615 genes enriched in Ncol3::mOrange2-positive cells (Figure S2; Table S1). We found that 268 out of the 944 DEGs in miR-2022 morphants overlapped with the list genes enriched in Ncol3::mOrange2-positive cells (Figure 2B). To test if such an overlap represented a significant enrichment, we simulated 10,000 lists of 944 randomly chosen genes from *Nematostella* transcriptomes, and tested the probability of getting as many as 268 genes overlapping with the list of cnidocyte-specific genes by chance (see STAR Methods). We found an average peak for 109.54 overlapping genes per list, with not even a single list containing as many as 268 cnidocyte-enriched genes (permutation test, $p = 0$, Figure 2C). Next, we analyzed a recently published *Nematostella* single-cell dataset[19] and examined which cell clusters contain the DEGs in Nv-miR-2022 morphants (Figure 2D). To this end, we generated a list of marker genes for each cell cluster resulting in 2,168 marker genes that are distributed between the 12 cell clusters. We collected all Nv-miR-2022 morphant DEGs that overlapped with the single-cell cluster markers and found that 65.4% of these genes (110/168) were in clusters 1 and 2 that correspond to cnidocytes, while the rest were spread between clusters 3–12. (Figure 2D). The majority of these DEGs were in cluster 2, which corresponds to mature cnidocytes. To test the significance of this enrichment we simulated 10,000 lists of random gene collections and never detected as many as 110 genes to overlap with cnidocyte clusters (permutation test, $p = 0$, average peak = 18.32 genes per list, Figure 2E). The downregulation of many cnidocyte-specific genes in our miR-2022 morphants (Figure 2) is in line with our observation of reduction of Ncol3::memOrange2-positive cells in these animals (Figure 1), further supporting the involvement of miR-2022 in regulation of cnidocyte biogenesis.

Hyl1b is a plant-like miRNA biogenesis factor that is neo-functionalized to control biogenesis of cnidocytes through controlling levels of Nv-miR-2022

Transcriptional regulators can be duplicated and then one of the paralogs can be recruited and neo-functionalized to facilitate the biogenesis of cnidocytes.[28] Previously we reported that the plant-like miRNA biogenesis factor, Hyl1La, which is broadly expressed in *Nematostella*, regulates the biogenesis of multiple miRNAs in *Nematostella*, including miR-2022.[11] Here, we focused on the paralog of Hyl1La, Hyl1Lb, which unlike the former is known to exhibit specific expression in cnidocytes.[10] We hypothesized that it could, therefore, be involved in the biogenesis of miR-2022. We designed a translation-blocking MO to knock down Hyl1Lb 4 days post injection of zygotes of WT females crossed to NvNcol3::memOrange2 males. Using stem-loop qPCR, we tested miR-2022 levels relative to four different abundant miRNAs that are not specific to cnidocytes: we observed a significant strong reduction only for Nv-miR-2022 but not for Nv-miR-2025, Nv-miR-2026, Nv-miR-2027, and Nv-miR-2028 (Figure 3A). This suggests that Hyl1b is essential for the biogenesis of miR-2022 in cnidocytes, but not for the other tested miRNAs. Similarly to Nv-miR-2022 morphants, we observed a significant reduction of glowing cnidocytes in Hyl1b morphants (Figure 3B). This effect was quantified and validated by sorting and counting Ncol3::memOrange2-positive cells from Hyl1b MO and control injected animals (t test, $p = 0.0318$, Figures 3C and S3B). Overall Hyl1Lb morphants mirrored the phenotype of Nv-miR-2022 morphants regarding its impact on cnidocytes, lowering their abundance without

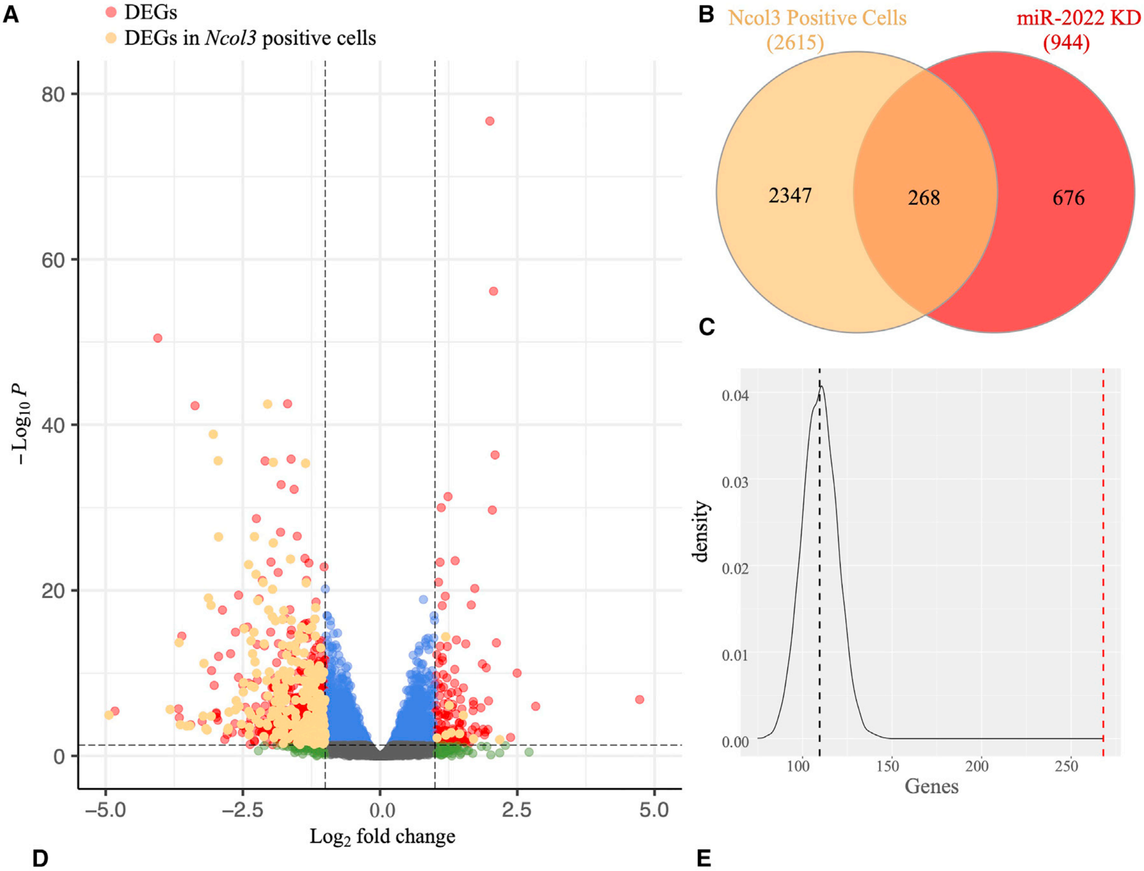

D

Cell-type	Cluster	DEGs
Cnidocyte	1	5
Mature cnidocyte	2	105
Gland mucin	3	1
Retractor muscle	4	19
Gastrodermis	5	18
Embryonic endomesoderm	6	2
Neuronal	7	4
Secretory	8	5
NPC	9	0
Ectoderm epidermis	10	5
Ectoderm embryonic	11	4
Ectoderm pharyngeal	12	0

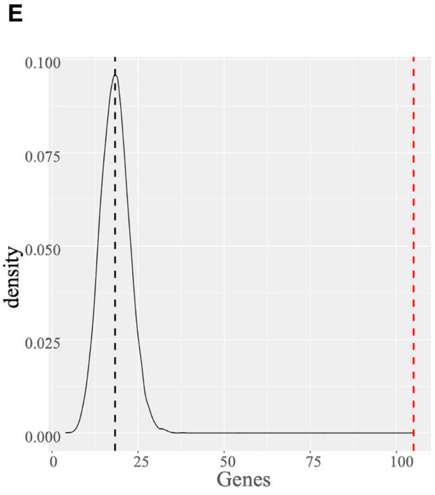

Figure 2. Transcriptome of *Nematostella* miR-2022 morphants

(A) Differentially expressed genes from 4-day-old planulae injected either with control or Nv-miR-2022 MOs. Differentially expressed genes (red and orange) were defined by FDR <0.05 and fold change ≥2. In orange: differentially expressed genes that are present in an independently obtained cnidocyte transcriptome (“Ncol3-positive cells”).[40]

(B) Overlapping portion of the differentially expressed genes identified in this study with genes from the cnidocyte transcriptomes.[40]

(C) The likelihood of 268 cnidocyte-specific genes overlapping with the list of 944 DEGs in miR-2022 morphants was assessed with Permutation test. 944 genes were randomly chosen 10,000 times (see STAR Methods for more details) and frequency of overlapping genes with genes of Ncol3-positive cells was plotted.

(D and E) Differentially expressed genes in miR-2022 morphants that are found within cell type markers of the previously published single-cell expression data,[19] and permutation test. Black lines in C and E represent the expected overlap by chance relative to the observed (red line).

CellPress
OPEN ACCESS

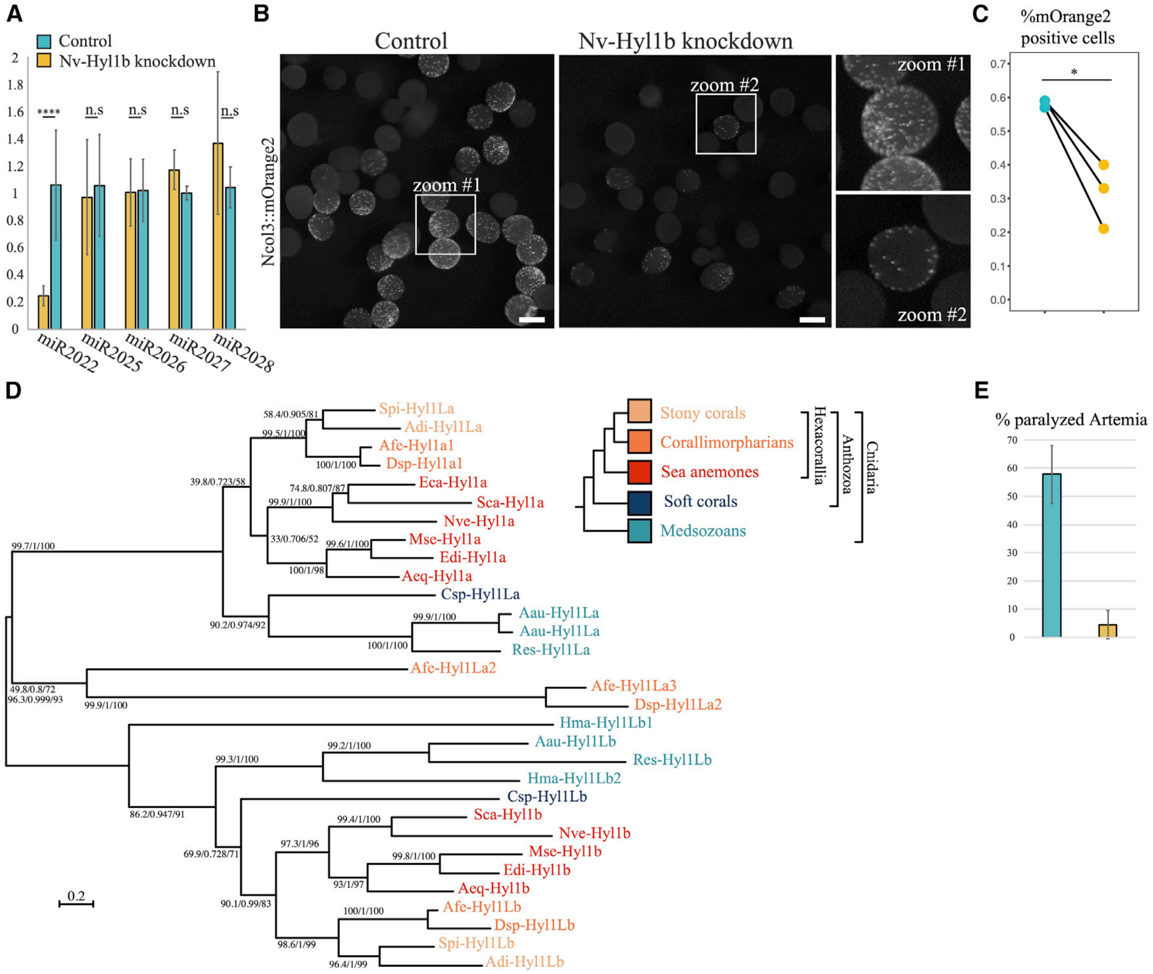

Figure 3. The impact of Nv-Hyl1b knockdown on cnidogenesis and prey paralysis potency in *Nematostella*

(A) Stem-loop qPCR assessment of Nv-miR-2022 levels as well levels of four additional non-cnidocyte miRNAs in *Nematostella* planulae 4 days post injection of control or Nv-Hyl1b MO into zygotes. Three independents biological replicates. Student's t test. p = 7.75E−05. Error bars represent standard deviation.

(B and C) (B) Intensity of mOrange2 in progeny of WT crossed with mOrange2::Ncol3 transgenes 4 days post injecting the zygotes with control or Nv-Hyl1b MO. Three independent biological replicates were subjected to flow cytometry analysis and the amounts of mOrange2-positive cells are quantified (C).

(D) Phylogenetic relationship of cnidarian Hyl1 homologs. Topology represents maximum likelihood consensus phylogenetic tree. Support values of the SH-aLRT (50–100), approximate Bayes test (0.6–1.0), and ultrafast bootstrap replicates (50–100) appear from left to right near each relevant node. Aau, *Aurelia aurita*; Adi, *Acropora digitifera*; Aeq, *Actinia equina*; Afe, *Amplexidiscus fenestrafer*; Csp, *Clavularia*; Dsp, *Discosoma*; Eca, *Edwardsiella_carnea*; Hma, *Hydra magnipapillata* (*vulgaris*); Mse, *Metridium senile*; Nve, *Nematostella vectensis*; Res, *Rhopilema esculentum*; Sca, *Scolanthus callimorphus*; Spi, *Stylophora pistillata*.

(E) Percentage of paralyzed *Artemia* 5.5 h post incubation with control or Nv-Hyl1b MO 4-day-old *Nematostella* planulae. Three independent biological replicates. Student's t test. p = 0.009112. Error bars represent standard deviation.

having a severe impact on the development (Figure S3A). This is in contrast to morphants of other miRNA biogenesis factors *in Nematostella* including Hyl1La, which block morphogenesis.[11,37,38] Interestingly, the ubiquitously expressed Hyl1La is essential for the biogenesis of multiple miRNAs including miR-2022.[11] However, in our Hyl1Lb knockdowns miR-2022 levels were abolished, suggesting that Hyl1La alone is sufficient for the biogenesis of multiple miRNA but not for miR-2022. Therefore, we conclude that the specificity of Hyl1Lb to biogenesis of Nv-miR-2022 stems from both their co-expression in cnidocytes and the specificity to process miR-2022 precursors.

To better understand the relation between miR-2022 and Hyl1Lb, we collected the sequences of Hyl1 homologs from multiple cnidarian species and constructed a new phylogeny (Figure 3D). The grouping of some of the homologs from both Anthozoa (sea anemones and corals) and Scyphozoa (true jellyfish), two distantly related cnidarian groups, with either Hyl1La or Hyl1Lb from *Nematostella* strongly suggests that Hyl1b originated through an ancient duplication and neo-functionalization event in the last common ancestor of all cnidarians.

Finally, as Hyl1Lb morphants mirror the reduction of cnidocytes through downregulation of Nv-miR-2022, we hypothesized that these morphants would exhibit defected capacity of predator paralysis similarly to Nv-miR-2022 morphants. Hence, we carried out the same paralysis assay presented in Figure 1E with Hyl1Lb 4-day-old morphants. Similarly to the assays with

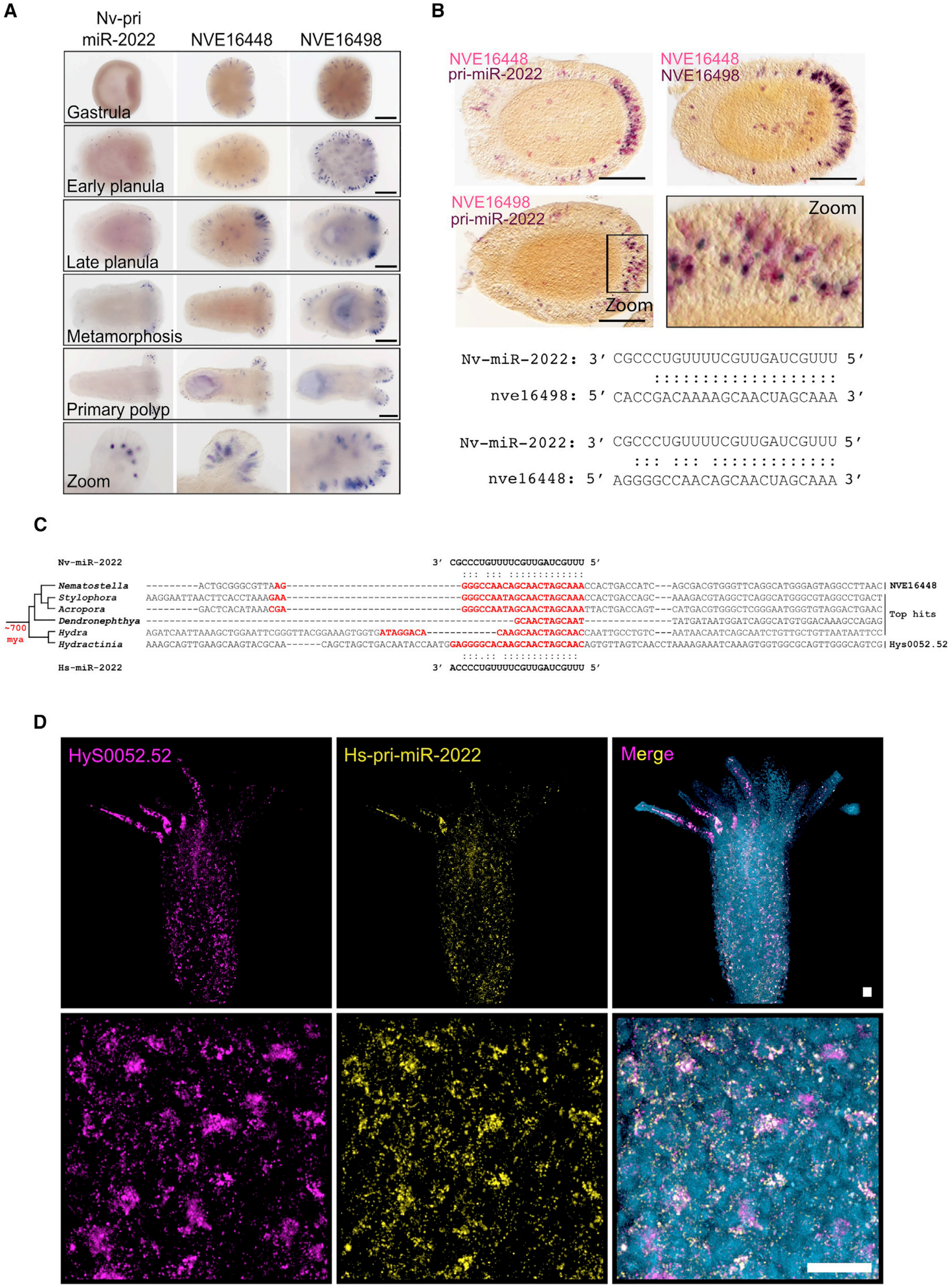

(legend on next page)

Nv-miR-2022 morphants, *Artemia* nauplii incubated with control injected *Nematostella* planulae showed a significantly higher number of paralyzed/dead individuals compared with *Artemia* incubated with Hyl1Lb morphants 5.5 h post incubation (t test, p = 0.009112, Figure 3E; Video S2).

Neo-functionalization of miRNA biogenesis/RISC proteins to specific tissues has been shown previously in bilaterians for Argonautes,[41] Dicers,[42] and other factors.[43] However, such events are mostly of recent origin and occur within distinct lineages of nematodes and vertebrates, not sharing an ancestral functional link to all bilaterians. In contrast, our phylogenetic analysis of Hyl1b strongly suggests that its recruitments to regulation of cnidogenesis occurred in the last cnidarian common ancestor (Figure 3D).

miR-2022 is expressed in the stinging cells in cnidarian species separated by >600 million years

Using *in situ* hybridization we tested if, in addition to *NR2*,[9] other targets exhibit overlapping expression with miR-2022 in cnidocytes of *Nematostella* (Figures 4A and 4B). To this end, we generated probes against the primary sequence of Nv-miR-2022 (pri-Nv-miR-2022) as well as against two additional targets: *NVE16448* and *NVE16498* (their target sites are cleaved by Nv-miR-2022 according to degradome sequencing[9]; Figure S4A). We found that both targets exhibited cnidocyte-specific expression and overlapped with pri-Nv-miR-2022 across early *Nematostella* development (Figures 4A and 4B). While *NVE16498* lacks a known function, *NVE16448* encodes a putative arylsulfatase (also known as chondroitinsulfatase). It was shown previously that non-sulfated chondroitin structurally stabilizes cnidarian cnidocysts,[44] raising a possible link between this enzyme and cnidocyte biogenesis.

Next, we computationally predicted targets of miR-2022 in several anthozoans (sea anemones and corals) and distantly related hydrozoan species. Surprisingly, a gene annotated as an arylsulfatase appeared in all species as one of the top targets. Reciprocal BLAST searches of this gene resulted in the predicted target of miR-2022 as the best hit in all species. This implies that homologs of NVE16448 are conserved targets of miR-2022 in Cnidaria (Figure 4C).

To further explore the functional basis for miR-2022 conservation, we used the publicly available single-cell RNA expression data of *Nematostella* and the hydrozoans *Hydra vulgaris* and *Hydractinia symbiolongicarpus*, which separated from Anthozoa at the dawn of cnidarian evolution. We found that the Arylsufatase-encoding gene with miR-2022 binding site (NVE16448, Hys0052.52, and t19037aep) was enriched in cnidocyte clusters of all three species (Figure S4). These results strongly suggest that regulation of the experimentally validated target, NVE16448, and the computationally predicted targets, Hys0052.52 and t19037aep, represent an ancestral regulation of an arylsulfatase-encoding gene by miR-2022 that persisted in extant cnidarians.

Finally, to test whether the expression of miR-2022 and its targets in cnidocytes is a cnidarian apomorphy, we characterized the expression patterns of Hys0052.52 and miR-2022 in *Hydractinia*. For this species, a protocol for signal amplification by exchange reaction fluorescence *in situ* hybridization (FISH) has been optimized and enables the use of shorter probes,[45] which in our hands are essential for localizing the pri-miRNA in this species. Using double-FISH combinations for *Hydractinia* pri-mir-2022 (Hs-pri-miR-2022), Minicollagen 1 (Ncol1, a known cnidocyte marker[29,46]), and Hys0052.52, we found that pri-miR-2022 in this species co-localized with its computationally predicted target Hys0052.52 (Figure 4D) as well as with the cnidocyte marker, and that the target overlaps with Ncol1 (Figure S4C).

Precision of gene expression output is often difficult to control using transcription factors alone because transcription is an inherently noisy process.[47] Therefore, complex biological processes that govern development and cell differentiation in bilaterians recruited miRNA regulation to tune levels of dosage-sensitive genes involved in these processes.[48–50] Two kinds of such miRNA-target modes of regulation are known: (1) coherent and (2) incoherent.[51] Coherent regulation can take part in biological processes such as promoting transitions of differentiating cells from their progenitors, by reciprocally inhibiting leftover transcripts belonging to the two opposing differentiated cell types.[52] In this type of regulation, miRNAs show little to no overlap in expression with their targets.[53] In incoherent regulation, which is rare in bilaterians, miRNAs exhibit spatial-temporal overlap with their targets. It may seem puzzling to simultaneously activate gene transcription with its post-transcriptional repressor. However, this mechanism was shown to enable reaching precise expression levels by setting a threshold that prevents the target from exceeding a certain level, regardless of its transcriptional intensity, enabling orchestration of precise stoichiometry of the targeted genes.[54] The co-expression of miR-2022 with experimentally validated (NVE16448, NVE 16498) and computationally predicted (Hys0052.52, t19037aep) targets in maturing cnidocytes of *Nematostella* and *Hydractinia* suggest that miR-2022 preserved its function of regulating cnidogenesis through incoherent regulation over 600 million years (Figures 4 and S4).

To date, only a handful of pan-cnidarian transcriptional regulators essential for cnidocyte biogenesis are known. Among them, transcription factors such as SoxB2 act upstream to

Figure 4. pri-miR-2022 with its targets in cnidocytes of *Nematostella* and *Hydractinia*

(A) Single ISH, NBT/BCIP staining using DIG-labelled probes for pri-miR-2022 and two of its targets across early developmental stages of *Nematostella*. Scale bars, 100 μm.

(B) Double ISH staining of pri-miR-2022 with its two targets in 4-day-old *Nematostella* planulae. Samples were sliced into 14 μm sections to reduce background staining noise. In the two upper panels, DIG-labelled probes and NBT/BCIP staining were used for pri-miR-2022 staining, and FITC-labelled probes and FastRed staining were used for the targets. In the second from bottom panel, DIG-labelled probes and NBT/BCIP staining, and FITC-labelled probes with FastRed staining were used for visualization of nve16498 and nve16448, respectively, Scale bars, 100 μm.

(C) Binding sites to miR-2022 on genes with top blast hits to nve16448 in distantly related cnidarians.

(D) *Hydractinia* signal amplification by exchange reaction FISH staining of Hs-pri-miR-2022 (yellow) and the predicted target Hys0052.52 (magenta).

cnidogenesis and impact differentiation of both neurons and cnidocytes,[17,45,55,56] and PaxA, which exhibits cnidocyte-specific expression in Hydrozoa and Anthozoa.[57,58] The specific co-expression of miR-2022 with its experimentally validated, and computationally predicted targets in *Hydractinia* and *Nematostella* (Figures 4 and S4), as well as the single-cell RNA expression data supporting cnidocyte-specific genes carrying binding sites for miR-2022 in *Nematostella*, *Hydra*, and *Hydractinia* strongly suggest that, on top of transcriptional regulation, post-transcriptional regulation by the highly conserved miR-2022 emerged in the last common ancestor of Cnidaria and played a crucial role in enabling the innovation of these novel cell types through regulation of their biogenesis. We suggest a model in which co-expression of miR-2022 with its targets sets a unified threshold for the targeted transcripts enabling their coordinated stoichiometry. Thus, while transcription factors played an essential role in the appearance and evolution of cnidocyte,[55–58] the complexity of the bursting organelle's biogenesis drove the evolution of a regulatory circuit that tuned the expression levels of such dosage-dependent cnidogenesis genes. To our knowledge, this is the first time the functional importance of an individual miRNA is demonstrated in a non-bilaterian animal.

Interestingly, most conserved bilaterian miRNA lack evidence to explain the functional basis for their conservation. One of the important exceptions is the conserved role of miR-1 in regulating adequate stoichiometry of multiple targets that assemble the highly complex V-ATPse complex in muscles of bilaterian animals.[6] Therefore, on a broader phylogenetic context, examples like miR-1 and miR-2022 exemplify how evolution utilized the post-transcriptional genetic toolkit to gain control of biogenesis of highly complex organelles.

To summarize, the emergence of cnidocytes at the dawn of cnidarian evolution required the acquisition of novel genes, rewiring the function of existing ones, and the emergence of complex regulatory mechanisms to orchestrate the timing and levels of gene expression to allow the formation of the highly complex stinging organelles. As a result of their efficiency in prey/predator paralysis, and their early origin, it is plausible that survival of cnidarians became highly dependent on these cells, resulting in a selective pressure to preserve them in members of all cnidarian lineages.[59]

In this work, we suggest that the evolution of the cnidarian-specific cell type, the cnidocyte, was accompanied by the emergence of miR-2022 as a novel regulator of its biogenesis. This could be facilitated by rewiring of the miRNA biogenesis factor Hyl1, following gene duplication, to regulate the levels of miR-2022 in these cells. Therefore, cnidocyte biogenesis is one of the most ancient conserved miRNA-regulated cellular processes in animals known to date.

Limitations of the study

In this study, we focused on testing the function of miR-2022 in early developing *Nematostella*. Interestingly, we were unable to obtain adult CRISPR mutants for miR-2022. Despite this limitation, our findings from transient miR-2022 knockdowns demonstrate a targeted loss of cnidocytes, accompanied by an inability to paralyze potential predators. These results demonstrate the physiological outcome for a cnidarian that would lose miR-2022, suggesting a functional basis for the conservation of miR-2022 over a period longer than 600 million years. Another significant observation from our study is that knockdown of Hyl1b phenocopies miR-2022 knockdown. At this stage, the molecular mechanism by which Hyl1b regulates miR-2022 levels remains unknown, which can be considered a limitation. However, as our data indicate Hyl1b specificity toward miR-2022 precursors it is an exciting candidate for future research to investigate the molecular mechanism that enables a general miRNA biogenesis factor to neo-functionalize in order to regulate the maturation of specific miRNAs.

STAR★METHODS

Detailed methods are provided in the online version of this paper and include the following:

- KEY RESOURCES TABLE
- RESOURCE AVAILABILITY
  - Lead contact
  - Materials availability
  - Data and code availability
- EXPERIMENTAL MODEL AND STUDY PARTICIPANT DETAILS
  - Animals
- METHOD DETAILS
  - *In situ* hybridizations (ISH and SABER-ISH)
  - Nv-miR-2022 and Nv-Hyl1b knockdowns
  - RNA sequencing
  - Stem-loop qPCR
  - Paralysis assay
  - Planulae dissociation and flow cytometry analysis
  - Prediction of miR-2022 targets
  - RNA sequencing analysis
  - Phylogenetic analysis

SUPPLEMENTAL INFORMATION

Supplemental information can be found online at https://doi.org/10.1016/j.celrep.2023.113072.

ACKNOWLEDGMENTS

The authors are grateful to Dr. Michal Bronstein and Ms. Adi Turjeman (The Center for Genomic Technologies of the Alexander Silberman Institute of Life Sciences, The Hebrew University) for their help with high-throughput sequencing. The authors would like to thank Dr. Uri Gat and Prof. Yehuda Tzfati (The Hebrew University) for granting access and guidance using their lab equipment. In addition, the authors would like to thank Dr. Grigory Genikhovich (University of Vienna) for material and protocol exchange during the early stages this project's establishment. A.F. was supported by EMBO Short Term Fellowship 8417 and Long Term Fellowship 914-2021 during parts of this work. This work was funded by the European Research Council Starting Grant (CNIDARIAMICRORNA, 637456) and Consolidator Grant (AntiViralEvo, 863809) to Y.M.

AUTHOR CONTRIBUTIONS

Y.M. and A.F. conceptualized the study and designed the experiments. A.F. carried out the experiments in *Nematostella* assisted by J.M.S. (RNA library preparation), I.K. (FACS), and A.M.T. (qPCR). In *Hydractinia*, experimental design was carried out by U.F. and M.S.-S., who performed the experiments.

E.C. contributed to experimental optimization. RNA sequencing data and single-cell expression data analysis was carried out by J.M.S. Y.M. and A.F. wrote the paper. All authors commented on and edited the text.

DECLARATION OF INTERESTS

The authors declare no competing interests.

Received: December 16, 2022
Revised: July 12, 2023
Accepted: August 18, 2023
Published: September 6, 2023

REFERENCES

1. Ameres, S.L., and Zamore, P.D. (2013). Diversifying microRNA sequence and function. Nat. Rev. Mol. Cell Biol. *14*, 475–488. https://doi.org/10.1038/nrm3611.
2. Bartel, D.P. (2018). Metazoan MicroRNAs. Cell *173*, 20–51. https://doi.org/10.1016/j.cell.2018.03.006.
3. Voinnet, O. (2009). Origin, biogenesis, and activity of plant microRNAs. Cell *136*, 669–687. https://doi.org/10.1016/j.cell.2009.01.046.
4. Brennecke, J., Stark, A., Russell, R.B., and Cohen, S.M. (2005). Principles of microRNA-target recognition. PLoS Biol. *3*, e85. https://doi.org/10.1371/journal.pbio.0030085.
5. Giraldez, A.J., Mishima, Y., Rihel, J., Grocock, R.J., Van Dongen, S., Inoue, K., Enright, A.J., and Schier, A.F. (2006). Zebrafish MiR-430 promotes deadenylation and clearance of maternal mRNAs. Science *312*, 75–79. https://doi.org/10.1126/science.1122689.
6. Gutiérrez-Pérez, P., Santillán, E.M., Lendl, T., Wang, J., Schrempf, A., Steinacker, T.L., Asparuhova, M., Brandstetter, M., Haselbach, D., and Cochella, L. (2021). miR-1 sustains muscle physiology by controlling V-ATPase complex assembly. Sci. Adv. *7*, eabh1434. https://doi.org/10.1126/sciadv.abh1434.
7. Caygill, E.E., and Johnston, L.A. (2008). Temporal regulation of metamorphic processes in Drosophila by the let-7 and miR-125 heterochronic microRNAs. Curr. Biol. *18*, 943–950. https://doi.org/10.1016/j.cub.2008.06.020.
8. Pandey, M., Bansal, S., Bar, S., Yadav, A.K., Sokol, N.S., Tennessen, J.M., Kapahi, P., and Chawla, G. (2021). miR-125-chinmo pathway regulates dietary restriction-dependent enhancement of lifespan in Drosophila. Elife *10*, e62621. https://doi.org/10.7554/eLife.62621.
9. Moran, Y., Fredman, D., Praher, D., Li, X.Z., Wee, L.M., Rentzsch, F., Zamore, P.D., Technau, U., and Seitz, H. (2014). Cnidarian microRNAs frequently regulate targets by cleavage. Genome Res. *24*, 651–663. https://doi.org/10.1101/gr.162503.113.
10. Moran, Y., Praher, D., Fredman, D., and Technau, U. (2013). The evolution of microRNA pathway protein components in Cnidaria. Mol. Biol. Evol. *30*, 2541–2552. https://doi.org/10.1093/molbev/mst159.
11. Tripathi, A.M., Admoni, Y., Fridrich, A., Lewandowska, M., Surm, J.M., Aharoni, R., and Moran, Y. (2022). Functional characterization of a 'plant-like' HYL1 homolog in the cnidarian Nematostella vectensis indicates a conserved involvement in microRNA biogenesis. Elife *11*, e69464. https://doi.org/10.7554/eLife.69464.
12. Grimson, A., Srivastava, M., Fahey, B., Woodcroft, B.J., Chiang, H.R., King, N., Degnan, B.M., Rokhsar, D.S., and Bartel, D.P. (2008). Early origins and evolution of microRNAs and Piwi-interacting RNAs in animals. Nature *455*, 1193–1197. https://doi.org/10.1038/nature07415.
13. Chapman, J.A., Kirkness, E.F., Simakov, O., Hampson, S.E., Mitros, T., Weinmaier, T., Rattei, T., Balasubramanian, P.G., Borman, J., Busam, D., et al. (2010). The dynamic genome of Hydra. Nature *464*, 592–596. https://doi.org/10.1038/nature08830.
14. Nong, W., Cao, J., Li, Y., Qu, Z., Sun, J., Swale, T., Yip, H.Y., Qian, P.Y., Qiu, J.W., Kwan, H.S., et al. (2020). Jellyfish genomes reveal distinct homeobox gene clusters and conservation of small RNA processing. Nat. Commun. *11*, 3051. https://doi.org/10.1038/s41467-020-16801-9.
15. Baumgarten, S., Cziesielski, M.J., Thomas, L., Michell, C.T., Esherick, L.Y., Pringle, J.R., Aranda, M., and Voolstra, C.R. (2018). Evidence for miRNA-mediated modulation of the host transcriptome in cnidarian-dinoflagellate symbiosis. Mol. Ecol. *27*, 403–418. https://doi.org/10.1111/mec.14452.
16. Liew, Y.J., Aranda, M., Carr, A., Baumgarten, S., Zoccola, D., Tambutté, S., Allemand, D., Micklem, G., and Voolstra, C.R. (2014). Identification of microRNAs in the coral Stylophora pistillata. PLoS One *9*, e91101. https://doi.org/10.1371/journal.pone.0091101.
17. Richards, G.S., and Rentzsch, F. (2014). Transgenic analysis of a SoxB gene reveals neural progenitor cells in the cnidarian Nematostella vectensis. Development *141*, 4681–4689. https://doi.org/10.1242/dev.112029.
18. Tourniere, O., Dolan, D., Richards, G.S., Sunagar, K., Columbus-Shenkar, Y.Y., Moran, Y., and Rentzsch, F. (2020). NvPOU4/Brain3 Functions as a Terminal Selector Gene in the Nervous System of the Cnidarian Nematostella vectensis. Cell Rep. *30*, 4473–4489.e5. https://doi.org/10.1016/j.celrep.2020.03.031.
19. Steger, J., Cole, A.G., Denner, A., Lebedeva, T., Genikhovich, G., Ries, A., Reischl, R., Taudes, E., Lassnig, M., and Technau, U. (2022). Single-cell transcriptomics identifies conserved regulators of neuroglandular lineages. Cell Rep. *40*, 111370. https://doi.org/10.1016/j.celrep.2022.111370.
20. Rachamim, T., Morgenstern, D., Aharonovich, D., Brekhman, V., Lotan, T., and Sher, D. (2015). The dynamically evolving nematocyst content of an anthozoan, a scyphozoan, and a hydrozoan. Mol. Biol. Evol. *32*, 740–753. https://doi.org/10.1093/molbev/msu335.
21. Lotan, A., Fishman, L., Loya, Y., and Zlotkin, E. (1995). Delivery of a nematocyst toxin. Nature *375*, 456. https://doi.org/10.1038/375456a0.
22. Holstein, T., and Tardent, P. (1984). An ultrahigh-speed analysis of exocytosis: nematocyst discharge. Science *223*, 830–833. https://doi.org/10.1126/science.6695186.
23. Nüchter, T., Benoit, M., Engel, U., Ozbek, S., and Holstein, T.W. (2006). Nanosecond-scale kinetics of nematocyst discharge. Curr. Biol. *16*, R316–R318. https://doi.org/10.1016/j.cub.2006.03.089.
24. Park, S., Piriatinskiy, G., Zeevi, D., Ben-David, J., Yossifon, G., Shavit, U., and Lotan, T. (2017). The nematocyst's sting is driven by the tubule moving front. J. R. Soc. Interface *14*, 20160917. https://doi.org/10.1098/rsif.2016.0917.
25. Meier, S., Jensen, P.R., Adamczyk, P., Bächinger, H.P., Holstein, T.W., Engel, J., Ozbek, S., and Grzesiek, S. (2007). Sequence-structure and structure-function analysis in cysteine-rich domains forming the ultrastable nematocyst wall. J. Mol. Biol. *368*, 718–728. https://doi.org/10.1016/j.jmb.2007.02.026.
26. Ozbek, S. (2011). The cnidarian nematocyst: a miniature extracellular matrix within a secretory vesicle. Protoplasma *248*, 635–640. https://doi.org/10.1007/s00709-010-0219-4.
27. Columbus-Shenkar, Y.Y., Sachkova, M.Y., Macrander, J., Fridrich, A., Modepalli, V., Reitzel, A.M., Sunagar, K., and Moran, Y. (2018). Dynamics of venom composition across a complex life cycle. Elife *7*, e35014. https://doi.org/10.7554/eLife.35014.
28. Sunagar, K., Columbus-Shenkar, Y.Y., Fridrich, A., Gutkovich, N., Aharoni, R., and Moran, Y. (2018). Cell type-specific expression profiling unravels the development and evolution of stinging cells in sea anemone. BMC Biol. *16*, 108. https://doi.org/10.1186/s12915-018-0578-4.
29. David, C.N., Ozbek, S., Adamczyk, P., Meier, S., Pauly, B., Chapman, J., Hwang, J.S., Gojobori, T., and Holstein, T.W. (2008). Evolution of complex structures: minicollagens shape the cnidarian nematocyst. Trends Genet. *24*, 431–438. https://doi.org/10.1016/j.tig.2008.07.001.
30. Balasubramanian, P.G., Beckmann, A., Warnken, U., Schnölzer, M., Schüler, A., Bornberg-Bauer, E., Holstein, T.W., and Özbek, S. (2012). Proteome of Hydra nematocyst. J. Biol. Chem. *287*, 9672–9681. https://doi.org/10.1074/jbc.M111.328203.

31. Karabulut, A., McClain, M., Rubinstein, B., Sabin, K.Z., McKinney, S.A., and Gibson, M.C. (2022). The architecture and operating mechanism of a cnidarian stinging organelle. Nat. Commun. *13*, 3494. https://doi.org/10.1038/s41467-022-31090-0.

32. Hwang, J.S., Takaku, Y., Momose, T., Adamczyk, P., Özbek, S., Ikeo, K., Khalturin, K., Hemmrich, G., Bosch, T.C.G., Holstein, T.W., et al. (2010). Nematogalectin, a nematocyst protein with GlyXY and galectin domains, demonstrates nematocyte-specific alternative splicing in Hydra. Proc. Natl. Acad. Sci. USA *107*, 18539–18544. https://doi.org/10.1073/pnas.1003256107.

33. Ha, M., and Kim, V.N. (2014). Regulation of microRNA biogenesis. Nat. Rev. Mol. Cell Biol. *15*, 509–524. https://doi.org/10.1038/nrm3838.

34. Kloosterman, W.P., Lagendijk, A.K., Ketting, R.F., Moulton, J.D., and Plasterk, R.H.A. (2007). Targeted inhibition of miRNA maturation with morpholinos reveals a role for miR-375 in pancreatic islet development. PLoS Biol. *5*, e203. https://doi.org/10.1371/journal.pbio.0050203.

35. Chen, C., Ridzon, D.A., Broomer, A.J., Zhou, Z., Lee, D.H., Nguyen, J.T., Barbisin, M., Xu, N.L., Mahuvakar, V.R., Andersen, M.R., et al. (2005). Real-time quantification of microRNAs by stem-loop RT-PCR. Nucleic Acids Res. *33*, e179. https://doi.org/10.1093/nar/gni178.

36. Zenkert, C., Takahashi, T., Diesner, M.O., and Özbek, S. (2011). Morphological and molecular analysis of the Nematostella vectensis cnidom. PLoS One *6*, e22725. https://doi.org/10.1371/journal.pone.0022725.

37. Fridrich, A., Modepalli, V., Lewandowska, M., Aharoni, R., and Moran, Y. (2020). Unravelling the developmental and functional significance of an ancient Argonaute duplication. Nat. Commun. *11*, 6187. https://doi.org/10.1038/s41467-020-20003-8.

38. Modepalli, V., Fridrich, A., Agron, M., and Moran, Y. (2018). The methyltransferase HEN1 is required in Nematostella vectensis for microRNA and piRNA stability as well as larval metamorphosis. PLoS Genet. *14*, e1007590. https://doi.org/10.1371/journal.pgen.1007590.

39. Love, M.I., Huber, W., and Anders, S. (2014). Moderated estimation of fold change and dispersion for RNA-seq data with DESeq2. Genome Biol. *15*, 550. https://doi.org/10.1186/s13059-014-0550-8.

40. Gahan, J.M., Kouzel, I.U., Jansen, K.O., Burkhardt, P., and Rentzsch, F. (2022). Histone demethylase Lsd1 is required for the differentiation of neural cells in Nematostella vectensis. Nat. Commun. *13*, 465. https://doi.org/10.1038/s41467-022-28107-z.

41. Seroussi, U., Lugowski, A., Wadi, L., Lao, R.X., Willis, A.R., Zhao, W., Sundby, A.E., Charlesworth, A.G., Reinke, A.W., and Claycomb, J.M. (2022). A Comprehensive Survey of C. elegans Argonaute Proteins Reveals Organism-wide Gene Regulatory Networks and Functions. Preprint at bioRxiv *2022*. https://doi.org/10.1101/2022.08.08.502013.

42. Flemr, M., Malik, R., Franke, V., Nejepinska, J., Sedlacek, R., Vlahovicek, K., and Svoboda, P. (2013). A retrotransposon-driven dicer isoform directs endogenous small interfering RNA production in mouse oocytes. Cell *155*, 807–816. https://doi.org/10.1016/j.cell.2013.10.001.

43. Dallaire, A., Frédérick, P.M., and Simard, M.J. (2018). Somatic and Germline MicroRNAs Form Distinct Silencing Complexes to Regulate Their Target mRNAs Differently. Dev. Cell *47*, 239–247.e4. https://doi.org/10.1016/j.devcel.2018.08.022.

44. Adamczyk, P., Zenkert, C., Balasubramanian, P.G., Yamada, S., Murakoshi, S., Sugahara, K., Hwang, J.S., Gojobori, T., Holstein, T.W., and Ozbek, S. (2010). A non-sulfated chondroitin stabilizes membrane tubulation in cnidarian organelles. J. Biol. Chem. *285*, 25613–25623. https://doi.org/10.1074/jbc.M110.107904.

45. Chrysostomou, E., Flici, H., Gornik, S.G., Salinas-Saavedra, M., Gahan, J.M., McMahon, E.T., Thompson, K., Hanley, S., Kilcoyne, M., Schnitzler, C.E., et al. (2022). A cellular and molecular analysis of SoxB-driven neurogenesis in a cnidarian. Elife *11*, e78793. https://doi.org/10.7554/eLife.78793.

46. Millane, R.C., Kanska, J., Duffy, D.J., Seoighe, C., Cunningham, S., Plickert, G., and Frank, U. (2011). Induced stem cell neoplasia in a cnidarian by ectopic expression of a POU domain transcription factor. Development *138*, 2429–2439. https://doi.org/10.1242/dev.064931.

47. Kepler, T.B., and Elston, T.C. (2001). Stochasticity in transcriptional regulation: origins, consequences, and mathematical representations. Biophys. J. *81*, 3116–3136. https://doi.org/10.1016/S0006-3495(01)75949-8.

48. Gutiérrez-Pérez, P., Santillán, E.M., Lendl, T., Schrempf, A., Steinacker, T.L., Asparuhova, M., Brandstetter, M., Haselbach, D., and Cochella, L. (2020). A Deeply Conserved miR-1 Dependent Regulon Supports Muscle Cell Physiology. https://doi.org/10.1101/2020.08.31.275644.

49. Pinzón, N., Li, B., Martinez, L., Sergeeva, A., Presumey, J., Apparailly, F., and Seitz, H. (2017). microRNA target prediction programs predict many false positives. Genome Res. *27*, 234–245. https://doi.org/10.1101/gr.205146.116.

50. Hornstein, E., and Shomron, N. (2006). Canalization of development by microRNAs. Nat. Genet. *38*, S20–S24. https://doi.org/10.1038/ng1803.

51. Ebert, M.S., and Sharp, P.A. (2012). Roles for microRNAs in conferring robustness to biological processes. Cell *149*, 515–524. https://doi.org/10.1016/j.cell.2012.04.005.

52. Stark, A., Brennecke, J., Bushati, N., Russell, R.B., and Cohen, S.M. (2005). Animal MicroRNAs confer robustness to gene expression and have a significant impact on 3'UTR evolution. Cell *123*, 1133–1146. https://doi.org/10.1016/j.cell.2005.11.023.

53. Shkumatava, A., Stark, A., Sive, H., and Bartel, D.P. (2009). Coherent but overlapping expression of microRNAs and their targets during vertebrate development. Genes Dev. *23*, 466–481. https://doi.org/10.1101/gad.1745709.

54. Bleris, L., Xie, Z., Glass, D., Adadey, A., Sontag, E., and Benenson, Y. (2011). Synthetic incoherent feedforward circuits show adaptation to the amount of their genetic template. Mol. Syst. Biol. *7*, 519. https://doi.org/10.1038/msb.2011.49.

55. Flici, H., Schnitzler, C.E., Millane, R.C., Govinden, G., Houlihan, A., Boomkamp, S.D., Shen, S., Baxevanis, A.D., and Frank, U. (2017). An Evolutionarily Conserved SoxB-Hdac2 Crosstalk Regulates Neurogenesis in a Cnidarian. Cell Rep. *18*, 1395–1409. https://doi.org/10.1016/j.celrep.2017.01.019.

56. Richards, G.S., and Rentzsch, F. (2015). Regulation of Nematostella neural progenitors by SoxB, Notch and bHLH genes. Development *142*, 3332–3342. https://doi.org/10.1242/dev.123745.

57. Babonis, L.S., and Martindale, M.Q. (2017). PaxA, but not PaxC, is required for cnidocyte development in the sea anemone Nematostella vectensis. EvoDevo *8*, 14. https://doi.org/10.1186/s13227-017-0077-7.

58. Siebert, S., Farrell, J.A., Cazet, J.F., Abeykoon, Y., Primack, A.S., Schnitzler, C.E., and Juliano, C.E. (2019). Stem cell differentiation trajectories in Hydra resolved at single-cell resolution. Science *365*, eaav9314. https://doi.org/10.1126/science.aav9314.

59. Erwin, D.H., Laflamme, M., Tweedt, S.M., Sperling, E.A., Pisani, D., and Peterson, K.J. (2011). The Cambrian conundrum: early divergence and later ecological success in the early history of animals. Science *334*, 1091–1097. https://doi.org/10.1126/science.1206375.

60. Genikhovich, G., and Technau, U. (2009). Induction of spawning in the starlet sea anemone Nematostella vectensis, in vitro fertilization of gametes, and dejellying of zygotes. Cold Spring Harb. Protoc. *2009*, pdb.prot5281. https://doi.org/10.1101/pdb.prot5281.

61. Frank, U., Nicotra, M.L., and Schnitzler, C.E. (2020). The colonial cnidarian Hydractinia. EvoDevo *11*, 7. https://doi.org/10.1186/s13227-020-00151-0.

62. Kishi, J.Y., Lapan, S.W., Beliveau, B.J., West, E.R., Zhu, A., Sasaki, H.M., Saka, S.K., Wang, Y., Cepko, C.L., and Yin, P. (2019). SABER amplifies FISH: enhanced multiplexed imaging of RNA and DNA in cells and tissues. Nat. Methods *16*, 533–544. https://doi.org/10.1038/s41592-019-0404-0.

63. DuBuc, T.Q., Schnitzler, C.E., Chrysostomou, E., McMahon, E.T., Febrimarsa, Gahan, J.M., Gahan, J.M., Buggie, T., Gornik, S.G., Hanley, S.,

CellPress
OPEN ACCESS

Barreira, S.N., et al. (2020). Transcription factor AP2 controls cnidarian germ cell induction. Science *367*, 757–762. https://doi.org/10.1126/science.aay6782.

64. Genikhovich, G., and Technau, U. (2009). In situ hybridization of starlet sea anemone (Nematostella vectensis) embryos, larvae, and polyps. Cold Spring Harb. Protoc. *2009*, pdb.prot5282. https://doi.org/10.1101/pdb.prot5282.

65. Moran, Y., Praher, D., Schlesinger, A., Ayalon, A., Tal, Y., and Technau, U. (2013). Analysis of soluble protein contents from the nematocysts of a model sea anemone sheds light on venom evolution. Mar. Biotechnol. *15*, 329–339. https://doi.org/10.1007/s10126-012-9491-y.

66. Varkonyi-Gasic, E., Wu, R., Wood, M., Walton, E.F., and Hellens, R.P. (2007). Protocol: a highly sensitive RT-PCR method for detection and quantification of microRNAs. Plant Methods *3*, 12. https://doi.org/10.1186/1746-4811-3-12.

67. Livak, K.J., and Schmittgen, T.D. (2001). Analysis of relative gene expression data using real-time quantitative PCR and the 2(-Delta Delta C(T)) Method. Methods *25*, 402–408. https://doi.org/10.1006/meth.2001.1262.

68. Sebe-Pedros, A., Saudemont, B., Chomsky, E., Plessier, F., Mailhe, M.P., Renno, J., Loe-Mie, Y., Lifshitz, A., Mukamel, Z., Schmutz, S., et al. (2018). Cnidarian Cell Type Diversity and Regulation Revealed by Whole-Organism Single-Cell RNA-Seq. Cell *173*, 1520–1534.e20. https://doi.org/10.1016/j.cell.2018.05.019.

69. Dai, X., Zhuang, Z., and Zhao, P.X. (2018). psRNATarget: a plant small RNA target analysis server (2017 release). Nucleic Acids Res. *46*, W49–W54. https://doi.org/10.1093/nar/gky316.

70. Bolger, A.M., Lohse, M., and Usadel, B. (2014). Trimmomatic: a flexible trimmer for Illumina sequence data. Bioinformatics *30*, 2114–2120. https://doi.org/10.1093/bioinformatics/btu170.

71. Lewandowska, M., Sharoni, T., Admoni, Y., Aharoni, R., and Moran, Y. (2021). Functional Characterization of the Cnidarian Antiviral Immune Response Reveals Ancestral Complexity. Mol. Biol. Evol. *38*, 4546–4561. https://doi.org/10.1093/molbev/msab197.

72. Putnam, N.H., Srivastava, M., Hellsten, U., Dirks, B., Chapman, J., Salamov, A., Terry, A., Shapiro, H., Lindquist, E., Kapitonov, V.V., et al. (2007). Sea anemone genome reveals ancestral eumetazoan gene repertoire and genomic organization. Science *317*, 86–94. https://doi.org/10.1126/science.1139158.

73. Dobin, A., Davis, C.A., Schlesinger, F., Drenkow, J., Zaleski, C., Jha, S., Batut, P., Chaisson, M., and Gingeras, T.R. (2013). STAR: ultrafast universal RNA-seq aligner. Bioinformatics *29*, 15–21. https://doi.org/10.1093/bioinformatics/bts635.

74. Li, B., and Dewey, C.N. (2011). RSEM: accurate transcript quantification from RNA-Seq data with or without a reference genome. BMC Bioinf. *12*, 323. https://doi.org/10.1186/1471-2105-12-323.

75. Haas, B.J., Papanicolaou, A., Yassour, M., Grabherr, M., Blood, P.D., Bowden, J., Couger, M.B., Eccles, D., Li, B., Lieber, M., et al. (2013). De novo transcript sequence reconstruction from RNA-seq using the Trinity platform for reference generation and analysis. Nat. Protoc. *8*, 1494–1512. https://doi.org/10.1038/nprot.2013.084.

76. Robinson, M.D., McCarthy, D.J., and Smyth, G.K. (2010). edgeR: a Bioconductor package for differential expression analysis of digital gene expression data. Bioinformatics *26*, 139–140. https://doi.org/10.1093/bioinformatics/btp616.

77. Schwaiger, M., Schönauer, A., Rendeiro, A.F., Pribitzer, C., Schauer, A., Gilles, A.F., Schinko, J.B., Renfer, E., Fredman, D., and Technau, U. (2014). Evolutionary conservation of the eumetazoan gene regulatory landscape. Genome Res. *24*, 639–650. https://doi.org/10.1101/gr.162529.113.

78. Liew, Y.J., Aranda, M., and Voolstra, C.R. (2016). Reefgenomics.Org - a Repository for Marine Genomics Data. Database (Oxford), 2016. https://doi.org/10.1093/database/baw152.

79. O'Leary, N.A., Wright, M.W., Brister, J.R., Ciufo, S., Haddad, D., McVeigh, R., Rajput, B., Robbertse, B., Smith-White, B., Ako-Adjei, D., et al. (2016). Reference sequence (RefSeq) database at NCBI: current status, taxonomic expansion, and functional annotation. Nucleic Acids Res. *44*, D733–D745. https://doi.org/10.1093/nar/gkv1189.

80. Edgar, R.C. (2004). MUSCLE: a multiple sequence alignment method with reduced time and space complexity. BMC Bioinf. *5*, 113. https://doi.org/10.1186/1471-2105-5-113.

81. Capella-Gutiérrez, S., Silla-Martínez, J.M., and Gabaldón, T. (2009). trimAl: a tool for automated alignment trimming in large-scale phylogenetic analyses. Bioinformatics *25*, 1972–1973. https://doi.org/10.1093/bioinformatics/btp348.

82. Nguyen, L.T., Schmidt, H.A., von Haeseler, A., and Minh, B.Q. (2015). IQ-TREE: a fast and effective stochastic algorithm for estimating maximum-likelihood phylogenies. Mol. Biol. Evol. *32*, 268–274. https://doi.org/10.1093/molbev/msu300.

83. Minh, B.Q., Nguyen, M.A.T., and von Haeseler, A. (2013). Ultrafast approximation for phylogenetic bootstrap. Mol. Biol. Evol. *30*, 1188–1195. https://doi.org/10.1093/molbev/mst024.

84. Anisimova, M., Gil, M., Dufayard, J.F., Dessimoz, C., and Gascuel, O. (2011). Survey of branch support methods demonstrates accuracy, power, and robustness of fast likelihood-based approximation schemes. Syst. Biol. *60*, 685–699. https://doi.org/10.1093/sysbio/syr041.

STAR★METHODS

KEY RESOURCES TABLE

REAGENT or RESOURCE	SOURCE	IDENTIFIER
Experimental models: Organisms/strains		
Nematostella vectensis	Moran lab	N/A
Hydractinia symbiolongicarpus	Frank lab	N/A
Chemicals, peptides, and recombinant proteins		
Nitro-blue tetrazolium, 5-bromo-4-chloro-3′-indolyphosphate (NBT/BCIP)	Roche	11681451001
Fast Red	Roche	11496549001
L-Cysteine	Merck Milipore	1028380100
TRIzol	Thermo Fisher Scientific	15596026
SuperScript III Reverse Transcriptase	Thermo Fisher Scientific	18080044
Fast SYBR Green Master Mix	Thermo Fisher Scientific	4385612
Calcein AM	Enzo	425203
Sytox blue	BioLegend	425305
Deposited data		
RNA-seq, Nv-miR-2022 morphants	This study	PRJNA893185
Oligonucleotides		
Nematostella. ISH. Nv-pri-miR-2022:	This paper	Table S2
Nematostella. ISH. Nve16498:	This paper	Table S2
Nematostella. ISH. Nve16448:	This paper	Table S2
Hydractinia (SABER-FISH). Hys0052.52 Hairpin 30. miRTarg2.30.1:	This paper	Table S2
Hydractinia (SABER-FISH). Hys0052.52 Hairpin 30.miRTarg2.30.3:	This paper	Table S2
Hydractinia (SABER-FISH). Hys0052.52 Hairpin 30. miRTarg2.30.4:	This paper	Table S2
Hydractinia (SABER-FISH). Hys0052.52 Hairpin 30.miRTarg2.30.8:	This paper	Table S2
Hydractinia (SABER-FISH). Hys0052.52 Hairpin 30. miRTarg2.30.10:	This paper	Table S2
Hydractinia (SABER-FISH). Hys-Mir2022 Hairpin 27. Pri-miR-2022.27.1:	This paper	Table S2
Hydractinia (SABER-FISH). Hys-MirNcol1 Hairpin 30. Ncol1.30.1:	This paper	Table S2
Hydractinia (SABER-FISH). Hys-MirNcol1 Hairpin 30. Ncol1.30.2:	This paper	Table S2
Hydractinia (SABER-FISH). Hys-MirNcol1 Hairpin 30. Ncol1.30.3:	This paper	Table S2
Hydractinia (SABER-FISH). Hys-MirNcol1 Hairpin 30. Ncol1.30.4:	This paper	Table S2
Hydractinia (SABER-FISH). Hys-MirNcol1 Hairpin 27. Ncol1.27.1:	This paper	Table S2
Hydractinia (SABER-FISH). Hys-MirNcol1 Hairpin 27. Ncol1.27.2:	This paper	Table S2
Hydractinia (SABER-FISH). Hys-MirNcol1 Hairpin 27. Ncol1.27.3:	This paper	Table S2
Hydractinia (SABER-FISH). Hys-MirNcol1 Hairpin 27. Ncol1.27.4:	This paper	Table S2
Nematostella. Morphoilino. Standard Control:	Gene Tools, LLC	N/A

(*Continued on next page*)

Continued

REAGENT or RESOURCE	SOURCE	IDENTIFIER
Nematostella. Morphoilino. miR-2022:	Gene Tools, LLC	N/A
Nematostella. Morphoilino. Hyl1lb. Translation blocking.	Gene Tools, LLC	N/A
Software and algorithms		
Trimmomatic	http://www.usadellab.org/cms/index.php?page=trimmomatic	N/A
		N/A
STAR	https://code.google.com/archive/p/rna-star/	N/A
RSEM	http://deweylab.biostat.wisc.edu/rsem	N/A
Trinity	https://github.com/trinityrnaseq/trinityrnaseq/wiki/Trinity-Differential-Expression	N/A
DESeq	http://www.bioconductor.org/packages/release/bioc/html/DESeq2.html	N/A
edgeR	https://bioconductor.org/packages/release/bioc/html/edgeR.html	N/A
EnhancedVolcano	https://github.com/kevinblighe/EnhancedVolcano	N/A
TrimAl	http://trimal.cgenomics.org/trimal	N/A
IQ-Tree	http://www.iqtree.org/	N/A

RESOURCE AVAILABILITY

Lead contact

Further information and requests for resources and reagents should be directed to and will be fulfilled by the lead contact, Yehu Moran (yehu.moran@mail.huji.ac.il).

Materials availability

This study did not generate new unique reagents.

Data and code availability

- Data corresponding to RNA-seq have been deposited to the NCBI Sequence Read Archive (SRA) with the bioproject ID: PRJNA893185. Any additional information required to reanalyze the data reported in this paper is available from the lead contact upon request.
- This paper does not report original code.

EXPERIMENTAL MODEL AND STUDY PARTICIPANT DETAILS

Animals

Nematostella polyps were grown in 16‰ sea salt water at 18°C. Polyps were fed with *Artemia salina* nauplii three times a week. Spawning of gametes and fertilization were performed according to a published protocol[60] as follows: In brief, temperature was raised to 25°C for 9 h and the animals were exposed to strong white light. Three hours after the induction, oocytes were mixed with sperm to allow fertilization.

Adult *Hydractinia symbiolongicarpus* colonies were maintained as described.[61] Specifically, female and male colonies were grown on glass slides kept in artificial seawater (ASW) at 19°C–22°C. Animals were fed four times per week with *Artemia* nauplii, and once a week with pureed oysters. To induce scheduled spawning, we kept the animals in a constant 14:10 light:dark cycle, where females and males spawn 1.5 h after exposure to light.

METHOD DETAILS

In situ hybridizations (ISH and SABER-ISH)

For *Hydractinia* polyps, Oligo probes were generated using hairpins 27 and 30.[62] Tissue fixation and dehydration followed by SABER-ISH were carried out as previously described.[45,63] Specifically, samples were incubated for 15 min in 4% $MgCl_2$ in 1:1 ration of

distilled water and filtered sea water, respectively. First fixation step was carried out for 90 s in fixation solution 1: Ice-cold 0.2% glutaraldehyde (stock: 25%, Sigma-Aldrich; G5882), 4% paraformaldehyde (stock: 16%, Alfa Aesar; 43368). Next samples were fixed for 1 h, 4°C in fixation solution 2: 4% paraformaldehyde in in PBS-0.1% Tween (PTW), followed by three quick washes of PTW. Samples were dehydrated in increasing concentrations of methanol in PTW and processed by incubation in 1% H_2O_2 diluted in 100% Ice-cold Methanol for 45 min. Next, samples were washed twice in ice-cold 100% MeOH, and permeabilized in 75% acetone in 25% methanol and rehydrated into PTW. Samples were then washed twice for 5 min with glycine in PTW (2 mg/mL) followed by three PTW washes at RT. Next, samples were washed three times with 1% (v/v) Triethanolamine pH 8.0 (TEA) in PTW for 5 min with 6 μL and 12 μL of acetic anhydride added to the second and third TEA washes, respectively. Next, samples were washed in PTW and incubated 10 min in pre-warmed Whyb buffer (2×SSC pH 7.0, 1% Tween 20, 40% Formamide) at 43C. At 43°C, Whyb buffer was replaced with Hyb1 buffer (2×SSC pH 7.0, 1% Tween 20, 40% Formamide, 10% Dextran sulfate) and incubated overnight (pre-hybridization). Samples were then transfered to Hybe buffer (5x SSC, 40% Formamide, 0.05 mg/mL Heparin, 0.25% Tween 20, 1% SDS, 1 mg/mL Roche blocking buffer powder, 1 mg/mL Salmon Sperm DNA) containing oligo probes and hybridized for two days at 43C. Next, probes were replaced with Whyb buffer for 10 min, followed by two 30 min washes with Whyb buffer, a 10 min wash with 50% Whyb buffer in 2x SSCTw (2×SSC pH 7.0, 0.1% Tween 20), and two 10 min washes with 2X SSCTw. Next, 2x SSCTw was replaced with two quick washes of PTW. Prewarmed Hyb2/fluor solution (1×PBS, 0.2% Tween 20, 10% Dextran sulfate/10 μM Fluor Oligo) was added to the samples (which were warmed up to 37C), for 1 h at 37C. The solution was replaced with pre-warmed Whyb2 (1×PBS, 0.1% Tween 20, 30% Formamide) for 10 min at 37C followed by two 5 min washes with PTW. Nuclear staining was performed at RT by using Hoechst dilution of 1:2000 in PTW and incubating for 45–60 min at RT. Samples were then washed twice with PTW and mounted in 97% TDE.The sequences of the probes are provided in the key resources table.

Colorimetric ISH and double ISH in *Nematostella* combining nitro-blue tetrazolium, 5-bromo- 4-chloro-3′-indolyphosphate (NBT/BCIP) and FastRed (Roche, Germany) staining was performed according to established published protocols[64,65] using the following DIG and FITC labeled probes (the sequences of the probes are available in the key resources table). Specifically, *Nematostella* larvae were fixed for 1.5 min in ice-cold 3.7% formaldehyde in 1/3 seawater with 0.2% glutaraldehyde, and then for 1 h in 3.7% formaldehyde in 1/3 seawater without glutaraldehyde. Next samples were washed extensively in PTW and stored in 100% methanol in −20°C. Next samples were rehydrated in increasing ratios of PTW/methanol, and washed 5 times in 100% PTW (5 min each). Samples were then digested with 0.01 mg/mL proteinase K in PTW for 20 min at RT without shaking. Digestion was stopped by two 5 min washes of 4 mg/mL glycine in PTW. Samples were washed 5 min in 0.1M triethanolamine in PTW, next again 2 more time with 3 μL and 6 μL per mL of acetic anhydride, respectively. Samples were then washed twice for 5 min in PTW and refixed in 3.7% formaldehyde in PTW for 1 h at RT. Next, the fixative was washed away with five washes of PTW (5 min each). Samples were then incubated for 10 min in 50%/50% PTW and hybridization solution (hybridization temperature), followed by 10 min incubation in hybridization solution. Next, prehybridization step was carried out by replacing the solution with fresh hybridization solution for 2 h. Probes were added to hybridization solution, denatured at 85°C for 5 min and added to the sample. Hybridization was then carried out at hybridization temperature over the weekend. Next, probes were washed away in decreasing concentrations of hybridization solution as follows: 10 min wash in hybridization solution, followed by 40 min wash in hybridization solution, followed by 30 min wash in 75% hybridization solution 25% 2X SSC, followed by 30 min wash 50% hybridization solution 50% 2X SSC, followed by 30 min wash 25% hybridization solution 75% 2X SSC, and finally in 100% 2X SSC for 30 min. Next samples were washed three times in 0.05X SSC, 20 min each. For detection, the following steps were carried out in room temperature: samples were re-introduced to PTW by three 30 min washes of increasing ratios of PTW/SSC 0.05X. Next, PTW was replaced with 5 washes of PBT (PBS 1X, 0.1% BSA, 0.2% Triton X-100), 5 min each. Samples were then blocked in blocking reagent for 1 h. Sheep anti-FITC coupled to alkalinephosphatase (Roche) was applied at a dilution of 1:2,000 in blocking reagent and incubated overnight at 4°C. Next, the samples were washed 10 times with PTW and were incubated in 0.1 M Tris–HCl (pH 8.2) for 5 min twice. Then FastRed reagent was applied in the same buffer. After appearance of a strong staining signal, the reaction was stopped by five quick washes followed by an inactivation of the enzyme by a single wash in 0.1 M glycine–HCl (pH 2.2) for 10 min at room temperature. After five additional washes in PTW, the samples were blocked for 1 h at RT with blocking reagent solution. Then sheep antiDIG alkaline-phosphatase-coupled antibody (Roche) was added at a concentration of 1:3,000 in blocking reagent and the samples were incubated overnight at 4°C. Next, the samples were washed ten times with PTW and nitroblue tetrazolium chloride (NBT)/5-bromo-4-chloro-3-indolylphosphate (BCIP) blue signal was developed as in the single *in situ* procedure (Genikhovich and Technau 2009). The staining was stopped by three washes in PTw and stained samples were mounted in 85% glycerol.

Nv-miR-2022 and Nv-Hyl1b knockdowns

Male adults of the Ncol3::memOrange2 transgenic line (generated in a previous study[28]) were spawned[60] and crossed with WT eggs. The gelatinous sack surrounding the eggs was removed using 4% L-Cysteine (Merck Milipore, USA) and followed by microinjecting the zygotes with antisense MOs. Next, zygotes were cultured at 22°C in 16‰ artificial seawater in the dark. The MO sequences were designed and synthesized by Gene Tools, LLC (USA) and their sequences are available in the key resources table. The Standard control MO was designed to have no matches to transcriptional start sites and splicing junctions of *Nematostella* transcripts, as well as not having matches to microRNA hairpins.

miR-2022 MOs (0.9mM), Hyl1b MO (0.45mM) and standard control MOs at equal concentrations were injected the same day into zygotes from the same batch to generate one biological replicate. In total, three biological replicates of ∼600 injected animals each

were generated for miR-2022 and control MO and subjected to morphological characterization, flow cytometry analysis and paralysis assays.

RNA sequencing

Total RNA was extracted using TRIzol Reagent (Thermo Fisher Scientific) and following manufacturer's protocol. Samples were treated with 2 μL of Turbo DNAse (Thermo Fisher Scientific) and undergoing an additional round of extraction using TRIzol Reagent. The total RNA quality was assessed using Bioanalyzer Nanochip (Agilent) with all samples having RNA Integrity Number (RIN) > 7.

Stem-loop qPCR

For the quantification of miRNAs, we used stem-loop primers[35] for miR-2022, miR-2025, miR-2026, miR-2027 and miR-2028 from our previous study.[11] For cDNA preparation, 100 ng of total RNA was reverse transcribed using the SuperScript III Reverse Transcriptase (Thermo Fisher Scientific). Specificity of miRNA primers was determined with endpoint PCR.[66] For this, we used 2 μL of cDNA as template, miRNAs-specific forward primer and stem-loop-specific reverse primer and run the PCR at 94°C for 2 min, followed by 35 cycles of 94°C for 15 s and 60°C for 1 min. For differential expression analysis, we ran qRT-PCR with 5sRNA as an internal control. For all the real-time experiments, we used Fast SYBR Green Master Mix (Thermo Fisher Scientific) and samples were run on StepOnePlus Real-Time PCR System (Thermo Fisher Scientific). Experiments were performed in three independent biological replicates and two technical replicates and data were analyzed using $2^{-\Delta\Delta Ct}$ method.[67]

Paralysis assay

4 days post injection with control, miR-2022 or HYl1b MOs, *Nematostella* planulae were incubated with *Artemia* in 0.5 mL *Nematostella* medium in 24 well plates. In each biological replicate ~60 planulae were divided into 3 wells (20 ± 3 per well) and incubated with 8–10 *Artemia* per well. Swimming *Artemia* were documented at time 0 and 5.5 h from the start of incubation. As a negative control, *Artemia* were incubated in *Nematostella* medium without planulae.

Planulae dissociation and flow cytometry analysis

Animals were dissociated into single cells as previously described.[68] Briefly, 4 days old planulae were placed in 1/3 strength calcium/magnesium free artificial seawater (17 mM of TrisHCl, 165 mM of NaCl, 3.3 mL of KCl and 9 mM of NaHCO3; final solution pH 8.0). Next, planulae were washed twice with 1/3 strength artificial seawater and incubated with 50 μg/mL liberaseTM (Roche) at 37°C for 10–20 min with occasional pipetting, until fully dissociated. About 200–500 individuals were used per tube. The reaction was stopped by adding 1/10 volume of 500 mM EDTA solution. Cells were centrifuged at 500× *g* at 4°C and resuspended in 1/3 strength artificial seawater containing 2 μg/mL calcein AM (Enzo) and 100 nM sytox blue (BioLegend) to monitor viability. The suspension was filtered using 5 mL round-bottom tubes with 35 μm cell strainer cap (Corning). Cells were incubated with the viability dyes for 20 min at room temperature and examined by fluorescence microscopy or flow cytometry.

For miR-2022 knockdowns, FACSAria III (BD Biosciences) equipped with 488-, 405- and 561-nm lasers was used to determine memOrange2 expression in single cells. Per run, 100,000 events were recorded. Sytox blue positive events were excluded and calceinAM positive events were included. FCS files were further analyzed using FlowJo V10 (BD Biosciences). For Hy1b knockdowns, CellStream (Merck) sorter was used using the same lasers, and 50,000 event were recorded.

Prediction of miR-2022 targets

Putative miRNA targets in *Hydra* and *Hydractinia* were identified with psRNATarget[69] with the following parameters: Maximum expectation score 2, GU penalty 0.5, extra weight seed 1.5, mismatches in seed 2, other mismatches 1, penalty for opening a gap 2, penalty for extending the gap 0.5, HSP size 19, seed region 2–13.

RNA sequencing analysis

Raw reads were trimmed and quality filtered by Trimmomatic,[70] using previously published parameters.[71] Reads were mapped to a modified *Nematostella*[72] which included an additional contig that encodes for memOrange2. Mapping was performed using STAR[73] and the gene counts quantified using RSEM.[74] Differential expression analyses were performed using scripts from Trinity[75] two both DESeq v2.1[39] and edgeR v3.16.[76] Gene models used in all downstream analyses were from previously published annotations (Schwaiger et al., 2014[77]; https://figshare.com/articles/Nematostella_vectensis_transcriptome_and_gene_models_v2_0/807696). Differentially expressed genes were defined by FDR <0.05 and fold change ≥2. Genes identified both methods were considered as differentially expressed. Biological replicates were quality checked for batch effect using sample correlation and principal component analysis. Volcano plots were generated using EnhancedVolcano (https://github.com/kevinblighe/EnhancedVolcano).

We then investigated the impact of miR-2022 knockdown on the transcriptional profile of nematocytes. To do this we performed comparative analysis using previously published RNAseq data,[40] generated from the primary polyps of a nematocyte transgenic reporter line, NvNcol3::mOrange2.[28] Raw reads were downloaded from the Sequence read archive (BioProject: PRJEB40304) for both NvNcol3::mOrange2 positive and negative cells and DGE analysis performed as previously described. To gain further insights into the expression of the targets of miR-2022 and other genes of interest at the cellular resolution, we investigated new scRNA-seq data across a time course of *Nematostella* development that was recently published Steger, J. et al. *2022.*[19] Counts matrix and cell

barcodes were downloaded from the Gene expression omnibus (accession: GSE200198) and scRNA-seq was performed using previously published methods (https://github.com/technau/CellReports2022/blob/main/DataS6_Merge2Figures.R).

We further tested whether genes significantly affected by miR-2022 KD are enriched in NvNcol3::mOrange2 positive cells as well as any specific cell cluster from scRNA-seq data across the development of *Nematostella*. To do this we performed permutation tests to validate that the overlap between genes differentially expressed following miR-2022 KD and those upregulated in NvNcol3::mOrange2 positive cells or marker genes of cell clusters are significant. Genes that had < one count per million for at least two libraries from the miR-2022 KD RNAseq data were removed to generate a background dataset. From this background, 944 genes were randomly selected 10,000 times and the overlap to genes upregulated in NvNcol3::mOrange2 positive cells and marker genes of cell clusters was compared.

Phylogenetic analysis

To construct the phylogenetic tree we selected representatives of HYL-1-Like sequences from anthozoans and medusozoans available in public databases. Specifically, *Nematostella* Hyl1a/b amino acid and nucleotide sequences were BLAST searched against protein and nucleotide databases of species present in the Reefgenomics database (*Acropora digitifera*, *Actinia equina*, *Amplexidiscus fenestrafer*, *Discosoma*, *Hydra magnipapillata (vulgaris)*, *Stylophora pistillata*).[78] The sequences Hyl1 from the rest of the species in the phylogeny (*Aurelia aurita*, *Clavularia*, *Edwardsiella_carnea*, *Metridium senile*, *Rhopilema esculentum*, *Scolanthus callimorphus*) are top BLAST hits against either Transcriptome Shotgun Assembly or RefSeq[79] databases of NCBI. The Hyl1s amino acid sequences were aligned using MUSCLE[80] and low quality alignment regions were removed by TrimAl[81] using the –automatic1 for heuristic model selection. The Maximum-Likelihood phylogenetic trees were constructed using IQ-Tree[82] with the VT + R3 model which was the best fitting model according to the Bayesian information criterion (BIC). Support values of the ML tree were calculated by three different methods: 1,000 ultrafast bootstrap replicates,[83] 1,000 replicates of the Shimodaira–Hasegawa approximate likelihood ratio test (SH-aLRT) and an approximate Bayes test.[84]

Cell Reports, Volume *42*

Supplemental information

An ancient pan-cnidarian microRNA regulates stinging capsule biogenesis in *Nematostella vectensis*

Arie Fridrich, Miguel Salinas-Saaverda, Itamar Kozlolvski, Joachim M. Surm, Eleni Chrysostomou, Abhinandan M. Tripathi, Uri Frank, and Yehu Moran

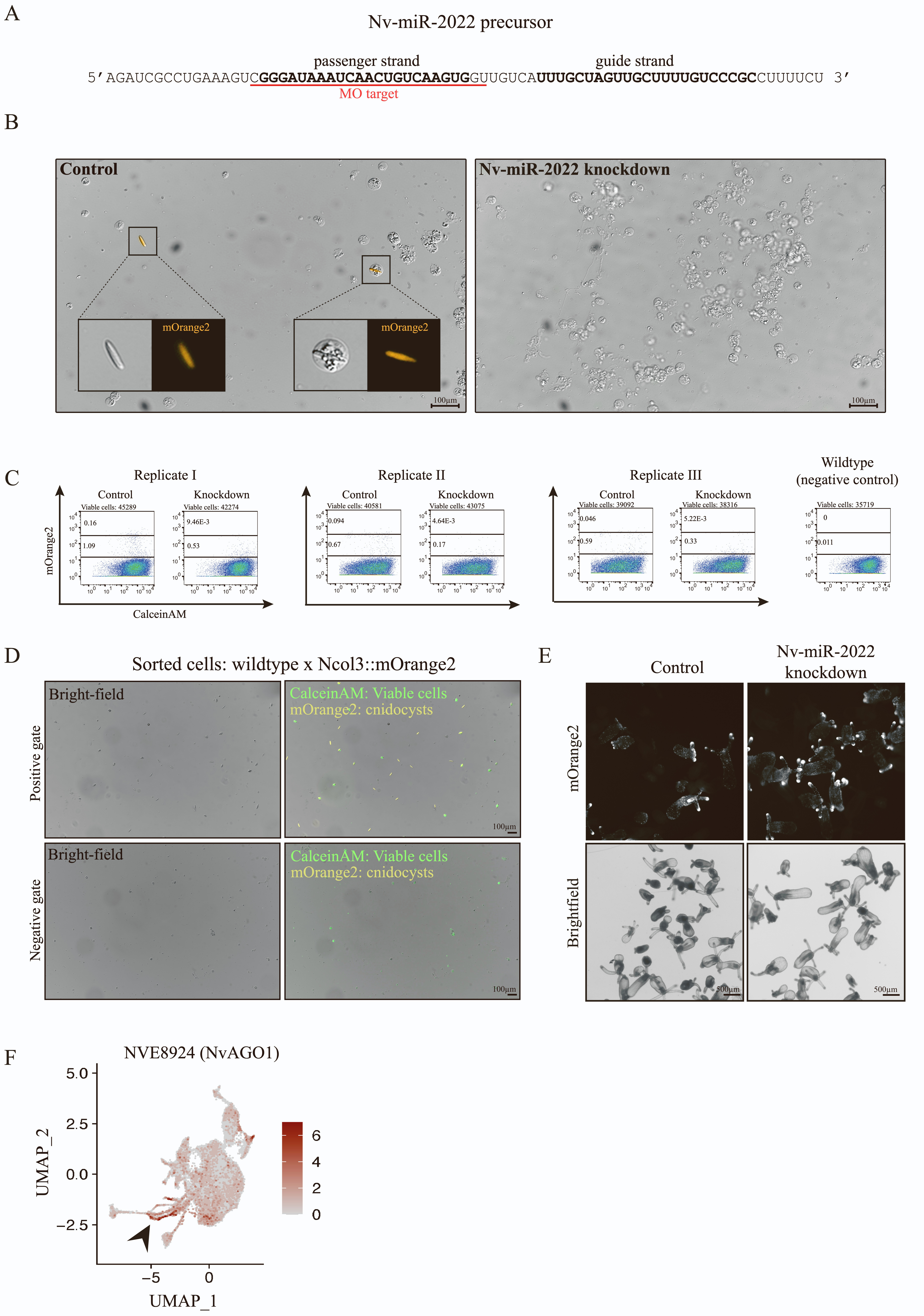
A
Nv-miR-2022 precursor
passenger strand
guide strand
5′AGAUCGCCUGAAAGUCGGGAUAAAUCAACUGUCAAGUGGUUGUCAUUUGCUAGUUGCUUUUGUCCCGCCUUUUCU 3′
MO target
B
Control
mOrange2
mOrange2
100µm
Nv-miR-2022 knockdown
100µm
C
Replicate I
Control
Knockdown
Viable cells: 45289
Viable cells: 42274
0.16
1.09
9.46E-3
0.53
mOrange2
CalceinAM
Replicate II
Control
Knockdown
Viable cells: 40581
Viable cells: 43075
0.094
0.67
4.64E-3
0.17
Replicate III
Control
Knockdown
Viable cells: 39092
Viable cells: 38316
0.046
0.59
5.22E-3
0.33
Wildtype
(negative control)
Viable cells: 35719
0
0.011
D
Sorted cells: wildtype x Ncol3::mOrange2
Positive gate
Negative gate
Bright-field
CalceinAM: Viable cells
mOrange2: cnidocysts
Bright-field
CalceinAM: Viable cells
mOrange2: cnidocysts
100µm
100µm
E
Control
Nv-miR-2022
knockdown
mOrange2
Brightfield
500µm
500µm
F
NVE8924 (NvAGO1)
UMAP_2
UMAP_1
5.0
2.5
0.0
−2.5
−5
0
6
4
2
0

Figure S1: The impact of miR-2022 knockdown on cnidogenesis and development of *Nematostella.* (**A**) Nv-miR-2022 precursor with highlighted region targeted by miR-2022 MO. (**B**) Dissociated cells of control and miR-2022 MO injected planulae 4 days post injection. Arrowheads mark cnidocytes. (**C**) Flow cytometry analysis of mOrange2::Ncol3 positive cells control and miR-2022 MO injected transgenes. Y axis represents the intensity of mOrange2 and cell viability is measured using CaleinAM on the X axis. Wildtype animals (right panel) were used set the threshold of the mOrange2 positive gate. Percentages of positive cells are indicates within the gates on the plot. (**D**) Cells from positive and negative gates were collected and visualized under a microscope to further confirm that glowing cells from the positive gates correspond to cnidocytes. (**E**) Intensity of mOrange2 and morphology of WT crossed with mOrange2::Ncol3 progeny 9 days post injecting the zygotes with control or miR-2022 MO. (**F**) Analysis of *Nematostella* single-cell RNA sequencing data generated by Steger, J. *et al.*[1] Arrowhead indicates cluster of cnidocytes and color intensity corresponds to expression intensities of NvAGO1.

A

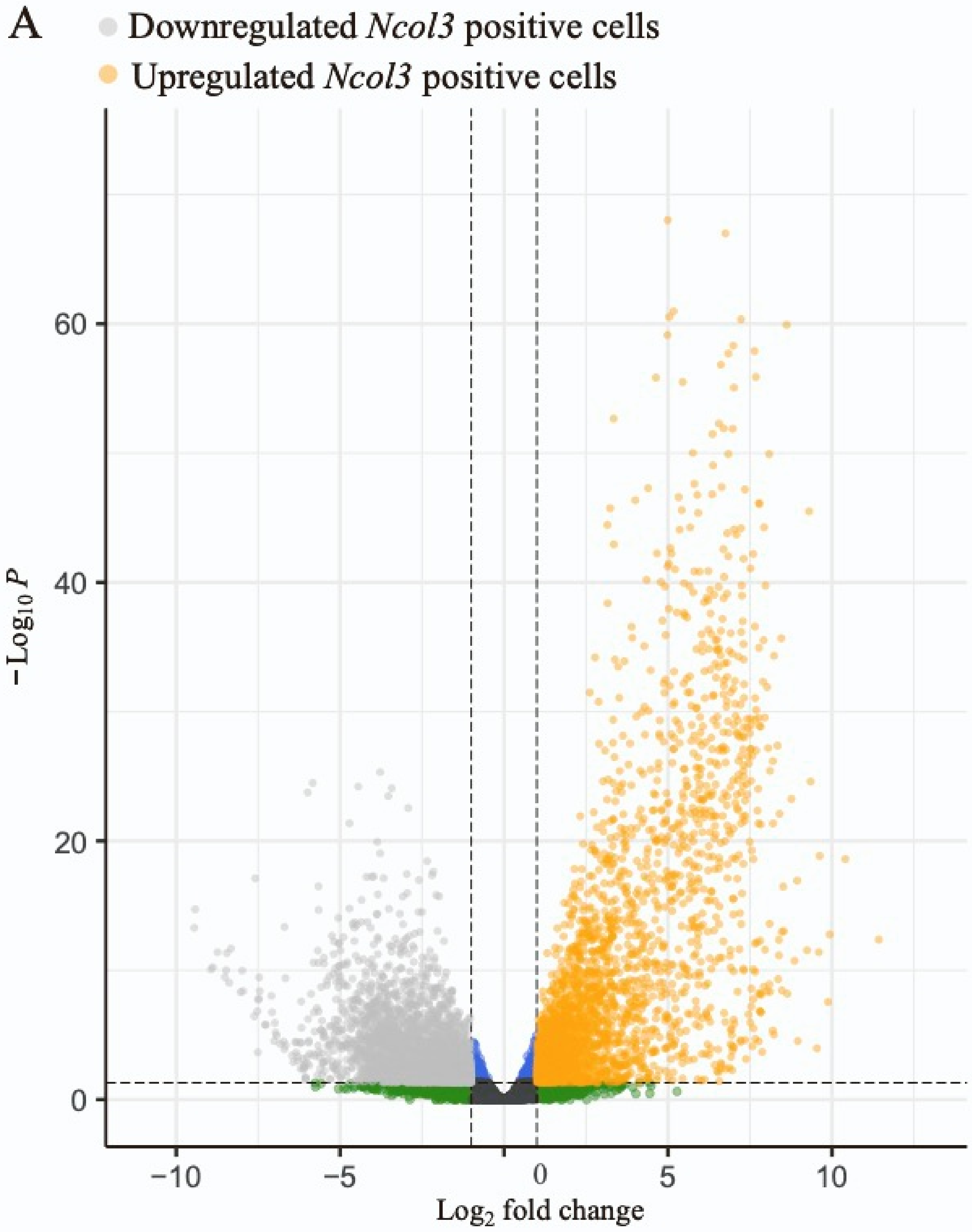

Downregulated *Ncol3* positive cells
Upregulated *Ncol3* positive cells
60
40
20
0
$-\text{Log}_{10} P$
−10
−5
0
5
10
Log_2 fold change

Figure S2: differential expression analysis to Identify cnidocyte specific genes in *Nematostella* primary polyps of Ncol3::mOrange2 transgenic line (see methods for more details).

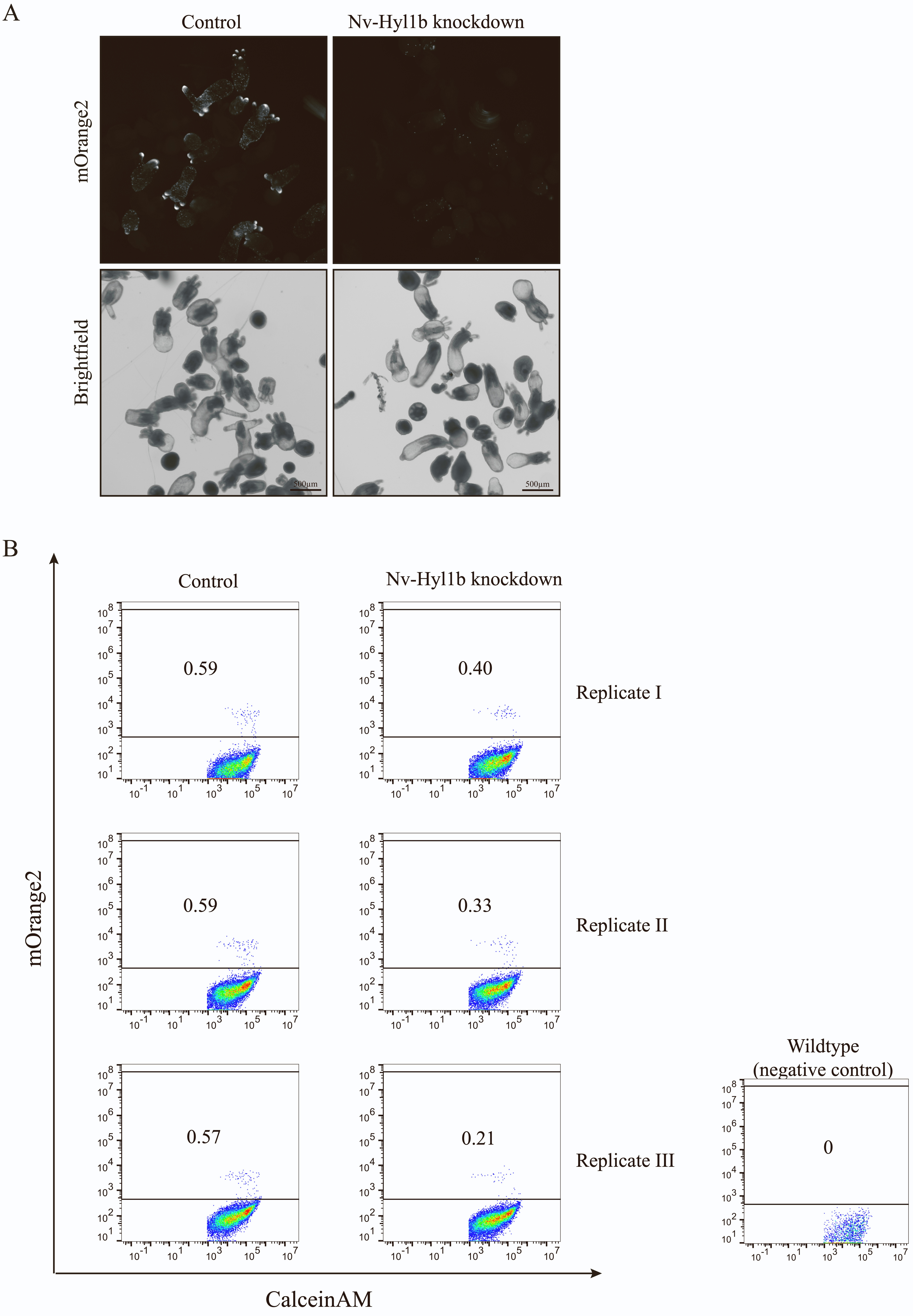
A
Control
Nv-Hyl1b knockdown
mOrange2
Brightfield
500µm
500µm
B
Control
Nv-Hyl1b knockdown
0.59
0.40
Replicate I
0.59
0.33
Replicate II
0.57
0.21
Replicate III
Wildtype
(negative control)
0
mOrange2
CalceinAM

Figure S3: The impact of Nv-Hyl1b knockdown on cnidogenesis and development of *Nematostella.* (**A**) Intensity of mOrange2 and morphology of WT crossed with mOrange2::Ncol3 progeny 9 days post injecting the zygotes with control or Nv-Hyl1b MO. (**B**) Flow cytometry analysis of mOrange2::Ncol3 positive cells control and Nv-Hyl1b MO injected transgenes. Y axis represents the intensity of mOrange2 and cell viability is measured using CaleinAM on the X axis. Wildtype animals (right panel) were used set the threshold of the memOrange2 positive gate. Percentages of positive cells are indicates within the gates on the plot.

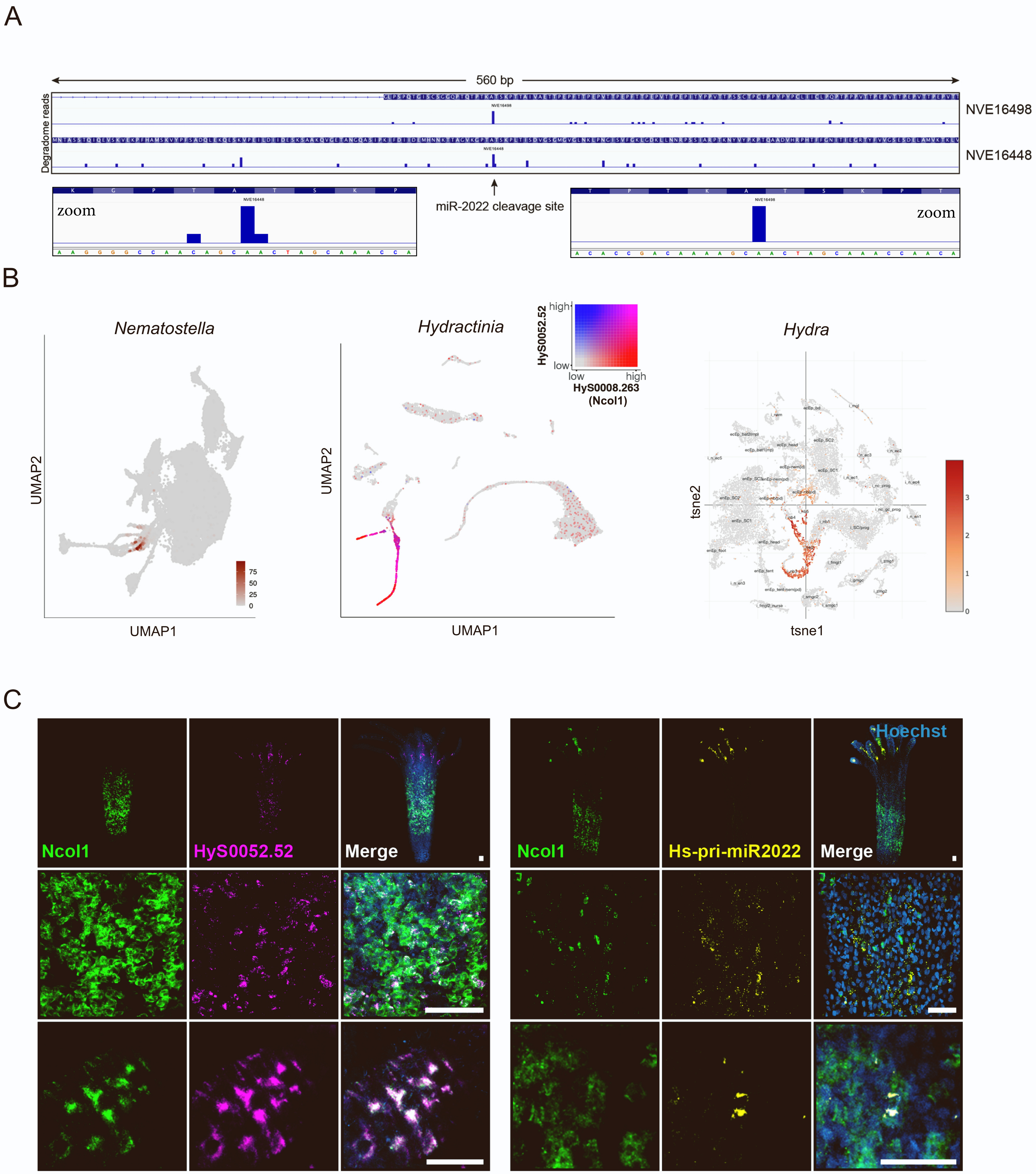
A
560 bp
Degradome reads
NVE16498
NVE16448
zoom
miR-2022 cleavage site
zoom
B
Nematostella
Hydractinia
Hydra
UMAP2
UMAP1
tsne2
tsne1
HyS0052.52
high
low
HyS0008.263
(Ncol1)
C
Ncol1
HyS0052.52
Merge
Ncol1
Hs-pri-miR2022
Merge
Hoechst

Figure S4: Expression of miR-2022 predicted targets in cnidocytes of *Hydra* and *Hydractinia*.(**A**) Degradome tracks spanning the experimentally validated Nv-miR-2022 targets NVE16498 and NVE16448 in *Nematostella*. (**B**) Single cells expressing NVE16448, Hys0052.52 and t19037aep in *Nematostella*, *Hydractinia* and *Hydra* (respectively). In *Hydractinia* expression intensities of Ncol1 and Hys0052.52 enable the visualization of the cnidocyte cluster of cells (bottom left) and the intensity of overlapping expression of Hys0052.52 within this cluster. In *Nematostella* the plot was generated as described in methods. In *Hydractinia* and *Hydra* plots were generated using the Single Cell Browser within the *Hydractinia* Genome Project Portal (https://research.nhgri.nih.gov/hydractinia) and using the *Hydra* single cell portal[2] (**C**) SABER FISH staining of Ncol1 (green) with Hys0052.52 (magenta) or with Hs-miR-2022 (yellow) in *Hydractinia* polyps.

1. Steger, J., Cole, A.G., Denner, A., Lebedeva, T., Genikhovich, G., Ries, A., Reischl, R., Taudes, E., Lassnig, M., and Technau, U. (2022). Single-cell transcriptomics identifies conserved regulators of neuroglandular lineages. Cell Rep *40*, 111370. 10.1016/j.celrep.2022.111370.
2. Siebert, S., Farrell, J.A., Cazet, J.F., Abeykoon, Y., Primack, A.S., Schnitzler, C.E., and Juliano, C.E. (2019). Stem cell differentiation trajectories in Hydra resolved at single-cell resolution. Science *365*. 10.1126/science.aav9314.
